# Proton Transport on Graphamine: A Deep-Learning Potential Study

**DOI:** 10.1021/acs.jpcc.5c05356

**Published:** 2025-11-14

**Authors:** Lakshmi Y. Ananthabhotla, Siddarth K. Achar, J. Karl Johnson

**Affiliations:** † Department of Chemical & Petroleum Engineering, 6614 University of Pittsburgh, Pittsburgh, Pennsylvania 15261, United States; ‡ Computational Modeling & Simulation Program, University of Pittsburgh, Pittsburgh, Pennsylvania 15260, United States

## Abstract

The performance of proton-exchange membrane fuel cells is critically dependent on the conduction of protons. Conventional proton exchange membranes employ materials such as Nafion that conduct protons only when properly hydrated. If the relative humidity is too low or too high, the fuel cell will cease to operate. This limitation highlights the need to develop new materials that can rapidly conduct protons without the need to regulate hydration. We present detailed atomistic simulations predicting that graphamine, which is an aminated graphane, conducts protons anhydrously with a very low diffusion barrier compared to existing materials. We have constructed a deep-learning framework tailored to modeling graphamine, enabling us to fully characterize and evaluate proton conduction within this material. The trained deep-learning potential is computationally economical and has near-density functional theory accuracy. We used our deep-learning potential to calculate the proton diffusion coefficients at different temperatures and to estimate the activation energy barrier for proton diffusion and found a very low barrier of 63 meV. We estimate the proton conductivity of graphamine to be 1322 mS/cm at 300 K. We show that protons hop along Grotthuss chains containing several amine groups and that the multidirectional hydrogen bonding network intrinsic in graphamine is responsible for the fast conduction of protons.

## Graphical abstract

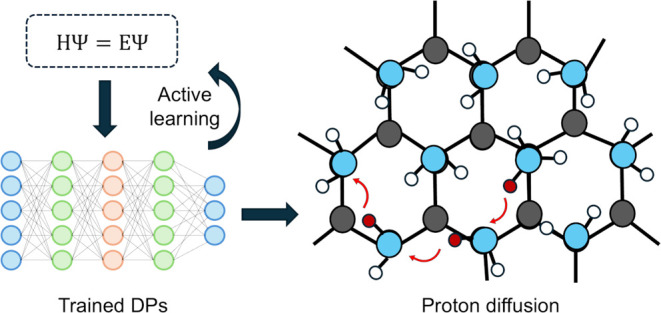

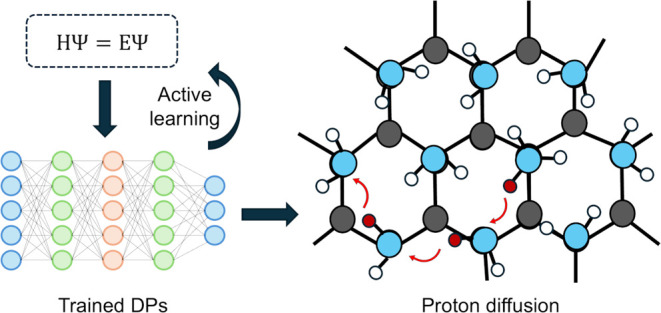

## Introduction

Proton exchange membrane (PEM) fuel cells are of critical importance in applications such as fuel cell electric vehicles and portable power generation.^1^ The advantages of fuel cells include high efficiency in the conversion of chemical energy to electrical energy and compact design compared to conventional combustion-based technologies.^2^ Due to these advantages, the growing demand for PEM fuel cells has driven an increasing interest in developing cost-effective and robust proton conducting materials to replace Nafion,^3–8^ which is a sulfonated fluoropolymer that is widely used as a membrane material in PEM fuel cells. A major drawback of Nafion is that it does not exhibit intrinsic proton conductivity and therefore requires adequate hydration to function effectively. This leads to water management issues, narrowing the operating temperature window to below about 80 °C, and requiring a complex design for Nafion-based PEM fuel cells.^9^ Developing intrinsically proton-conducting materials capable of operating anhydrously over an intermediate temperature range, 100–300 °C,^10^ can effectively circumvent these limitations and provide significant advantages.^10–13^

In the last few decades, a wide range of new materials have been proposed and studied for facilitating anhydrous or low-humidity proton conductivity. These include organic polymer electrolytes,^14,15^ molecular porous materials,^16^ porous organic frameworks,^17^ metal–organic framework electrolytes,^18^ covalent organic frameworks,^19–21^ nonmetal–organic frameworks,^22^ metallopolymers^23^ and solid acid electrolytes.^24^ However, these existing materials have various limitations,^25,26^ such as degrading under acidic/humid conditions, complex synthesis requirements, low intrinsic proton conductivity, etc. Hence, there is a need to develop new materials to enable cost-effective and reliable anhydrous proton conductivity in PEM fuel cells.

In this work, we use atomistic simulation methods to characterize proton conductivity on graphamine, which is graphane (sp^3^ hydrogenated graphene) functionalized with amine groups. Our study of graphamine is motivated by previous density functional theory (DFT) predictions by Bagusetty et al., indicating that graphamine is capable of conducting protons anhydrously.^27^ They observed proton diffusion at 1000 K from DFT-molecular dynamics (DFT-MD) simulations. However, because of the high computational demand of DFT, they were not able to compute diffusion coefficients at lower temperatures. They observed proton hopping along the 2-D network of hydrogen bonds formed by the amine groups, as shown in Figure 1. However, they were not able to fully characterize the proton transport mechanism, also due to computational limitations. Furthermore, the method that Bagusetty et al. used to estimate the diffusion coefficient relied on calculating the mean squared displacement of all the hydrogen atoms bonded to N atoms. This approach underestimated the actual proton diffusivity by overcounting the number of mobile species. Therefore, the goals of this work include an accurate estimate of the proton diffusion coefficients on graphamine as a function of temperature and elucidation of the proton conduction mechanism.

**Figure 1:**
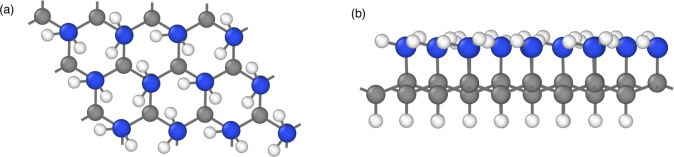
(a) Top-view and (b) side-view of a graphamine unit cell containing 24 carbon, 12 nitrogen and 36 hydrogen atoms. Note that the hydrogen atoms bound to N atoms are hydrogen bonded to neighboring N atoms, providing a percolating pathway for proton hopping. Blue: N atoms, gray: C atoms, white: H atoms.

Given the computational limitations of DFT and other accurate quantum chemical methods, we have chosen to use machine learning to develop atomistic potentials to study the dynamic properties of protonated graphamine. Specifically, we developed deep-learning potentials (DPs) using the DeePMD formalism^28^ to train DPs having near-DFT accuracy and efficient linear-scaling computational performance.

Another goal of this work is to compare the diffusivities, conductivities, and the proton transport mechanism for graphamine to a related material, hydroxylated graphane, or graphanol.^29–32^ A key difference between graphamine and graphanol is the number of hydrogen bonds the amine or hydroxyl group can make with neighboring moieties. The amine groups have a higher number of potential hydrogen bonds, and hence more possible directions for proton hopping.

It is important to address the feasibility of synthesizing graphamine at the outset, since it is not useful to predict the properties of materials that cannot be synthesized in practice. Different forms of aminated graphane have been synthesized in the past few years.^33–35^ These studies mainly focus on functionalizing graphene instead of graphane. Ideally, graphamine is expected to have all H atoms of graphane replaced with NH_2_ groups, but experimentally synthesized materials can have defects. We expect that functionalized graphamine with defects (e.g., missing NH_2_ groups) will conduct protons and follow a similar proton hopping mechanism presented in this study, since the percolation threshold for graphamine is the same as for graphanol, which is 0.5 (i.e., a material having only 50% of possible sites functionalized will still conduct protons).^31^

Our calculations show that protonated graphamine exhibits extremely high proton conductivity. We estimate a value of 1322 mS/cm at 300 K, which is higher than any other material of which we are aware. Analysis of the temperature dependence of the calculated proton diffusion coefficients gives a very low apparent activation energy barrier of *E*_*A*_ = 63 meV. We show that graphamine has a higher conductivity and lower diffusion barrier compared with graphanol and we identify the difference in hydrogen bonding networks as being responsible for graphamine’s enhanced performance.

## Computational Methodology

### DFT Methods

DFT calculations were performed to optimize the unit cells of graphamine, generate training data for the DPs, and produce reference data to validate the DPs. The Vienna ab initio simulation package (VASP)^36–39^ was used to perform all DFT calculations. Electron–ion interactions were described using the projected augmented-wave method.^40^ A kinetic energy cutoff for the plane-wave expansion of 400 eV was used. We used the revised Perdew–Burke–Ernzerhof (RPBE)^41,42^ generalized gradient approximation exchange-correlation functional and added the DFT-D3 van der Waal dispersion correction with the Becke-Johnson damping function.^43^ This choice was based on comparing binding energies for various hydrogen bonded NH_3_ dimer geometries,^44^ which we computed from CCSD­(T) at the complete basis set limit using the ORCA software package,^45^ with binding energies computed from VASP using different combinations of exchange-correlation functionals and van der Waals dispersion corrections. The total energy convergence for self-consistent field calculations was set to 10^–6^ eV. The ionic positions were relaxed until the forces were lower than the tolerance of 10^–3^ eV/Å for optimization calculations. Since graphamine is an insulator, we used an ISMEAR value of −5. All molecular dynamics calculations performed used the Nosé-Hoover thermostat.^46^

We considered a graphamine system composed of 24 C atoms with amine groups on only one side of the graphane. In experiments, both sides of the graphane would be functionalized. We only consider single-sided graphamine for computational convenience. Functionalizing both sides is not expected to change the results of our work, as shown by previous work on graphanol.^32^ We used the following naming conventions in our studies of protonated and nonprotonated systems. The protonated systems contain a single proton. A “n24C” system is a nonprotonated system consisting of 24 C atoms, 12 N atoms, and 36 H atoms. Similarly, a “p24C” system is a protonated system containing 24 C atoms, 12 N atoms and 37 H atoms, but having the same number of electrons as the n24C system. A n24C cell was transformed into a p24C cell by the introduction of a proton close to one of the NH_2_ groups, giving a system with a +1 e charge. Calculations on charged systems under periodic boundary conditions were handled with a homogeneous neutralizing background jellium charge. This background charge was shown to have a very small influence on proton dynamics for graphanol.^29^

### DP Training

We used the DeePMD-kit^28,47^ software to train DPs for graphamine. A schematic of DP training and evaluation is shown in Figure 2. The training protocol follows a similar approach to that described by Achar et al.,^31^ originally developed for graphanol. Details of the architecture of DPs are given in the .

**Figure 2:**
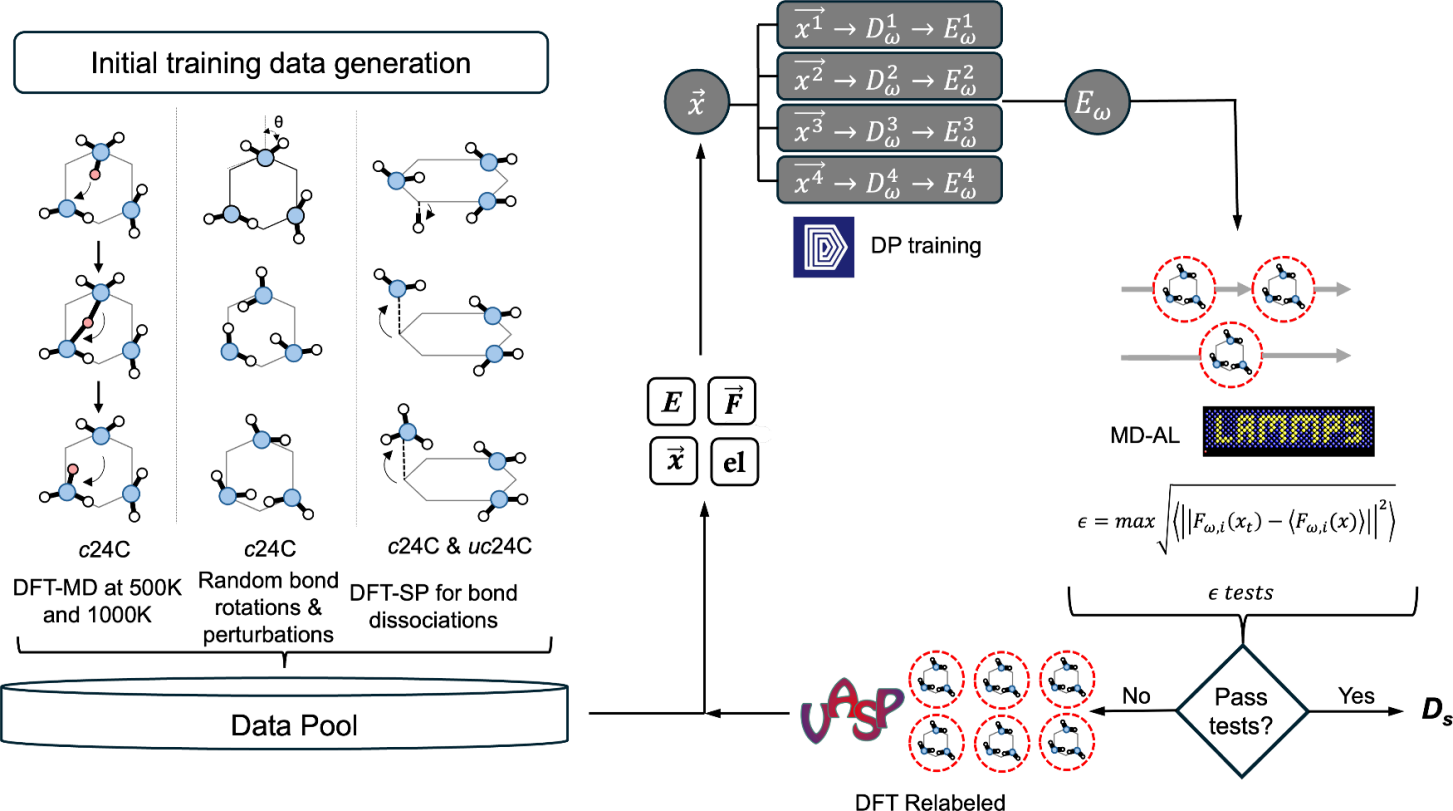
DP-AL training schematic. First, initial training data generated using p24C and n24C systems. Three main sets of initial data was generated based on proton hopping events, random NH_2_ group rotations and C–H, C–NH_3_ bond-dissociation events. An ensemble of four DPs (represented as *E*_ω_) was developed to perform data relabeling using MD exploration. The DP was accepted if the convergence criterion was reached, otherwise filtered configurations from the MD exploration were relabeled with DFT and added back to the training data set.

Initial training data were generated to capture key aspects of graphamine: (1) proton conduction, (2) amine group rotation, and (3) amine group stability during proton conduction. The sampling of proton conduction events was achieved by performing *NVT* DFT-MD simulations at *T* = 500, 1000, and 1200 K for 12.5 ps with a time step of 0.25 fs, using the n24C and p24C systems. Additional *NVT* DFT-MD simulations at *T* = 1000 K and 0.25 ps (0.25 fs timesteps) were conducted to sample amine group rotations, initialized with randomized torsional angles that describe the rotation of the NH_2_ groups. We note that we have not included room temperature data in our training data set even though it is important for the DP to be able to accurately model events around 300 K. It has been shown previously that training a DP using higher temperature DFT-MD produces accurate results for low temperatures, but the converse is not true.^48^

We accounted for C–N and C–H bond dissociation events in graphamine, as described in the . DFT single-point energies were computed for each structure and added to the training data set. We also sampled structures by perturbing the lattice parameters in the input file to sample compression and expansion of the graphamine lattice and performed DFT single-point energy calculations and added these to the initial training set. The initial training data set included 8000 snapshots in total.

This data set served as the starting point for an active learning cycle using the DP-GEN framework,^49^ which automates DP refinement through iterative exploration of the potential energy surface. In each iteration, an ensemble of four DPs was trained using random initializations of the neural network model parameters. One of the DPs was selected to run LAMMPS-MD (Large-scale Atomic/Molecular Massively Parallel Simulator)^50^ simulations. Model uncertainties were estimated by computing the maximum force deviations on the MD trajectories with all four DPs in the ensemble. These configurations were filtered and relabeled for DFT single-point energy calculations. These structures were then incorporated into the training set for the next round. This process was repeated until convergence was achieved (see for more details). After convergence, the DP with the lowest energy loss function value was used as the final DP for studying proton conduction on graphamine.

### Center of Excess Charge

We used the center of excess charge (CEC) method to compute proton mean squared displacements. The CEC approach estimates the position of the proton by identifying the positions of N atoms bonded to three nearest neighbor H atoms, which serve as the position of local charge centers (*r*_CEC_). The CEC method was originally developed and validated by Li and Swanson^51^ for modeling proton diffusion in bulk water and later modified by Achar et al.^31^ to study proton diffusion in graphanol. We adapted that implementation to model proton transport in graphamine. More details regarding the CEC method are given in the . We tracked the CECs to estimate the mean squared displacements and hence the self-diffusivities of protons (*D*_*S*_). We estimated *D*_*S*_ from the mean squared displacement using the Einstein relation. We used the Arrhenius equation to estimate the proton transport activation energy barrier,

1 $$\ln\left(D_{S}\right)=\ln\left(D_{0}\right)-\frac{E_{A}}{RT}$$

from simulations at temperatures of *T* = 300, 350, 400, and 500 K. We compared our estimated value of *E*_*A*_ for graphamine with that of graphanol.

## Results and Discussion

### DP Accuracy

Before studying the proton transport on graphamine, it is imperative to validate the accuracy of our DP. As a first assessment of the DP, we generated two sets of testing data, unseen by the trained DPs. These data include five independent *NVT* DFT-MD simulations at 1000 K with the p24C system and three independent *NVT*-MD simulations at 600, 800, and 1000 K using the n24C system. All the *NVT* DFT-MD simulations were 12.5 ps long. Each of these sets of test data contained 3000 snapshots. We used these testing data to make four parity plots. Parity plots of the energies and forces predicted from the DPs are given in Figure 3. We report the root mean squared error (RMSE) in predictions for each case in these plots. The RMSE in predicted energies are 2.19 × 10^–3^ and 1.59 × 10^–3^ eV/atom for the nonprotonated and protonated systems, respectively. These values are within chemical accuracy of 0.04 eV. The corresponding RMSE in predicted forces are 0.125 and 0.105 eV/Å, for the n24C and p24C systems, respectively.

**Figure 3:**
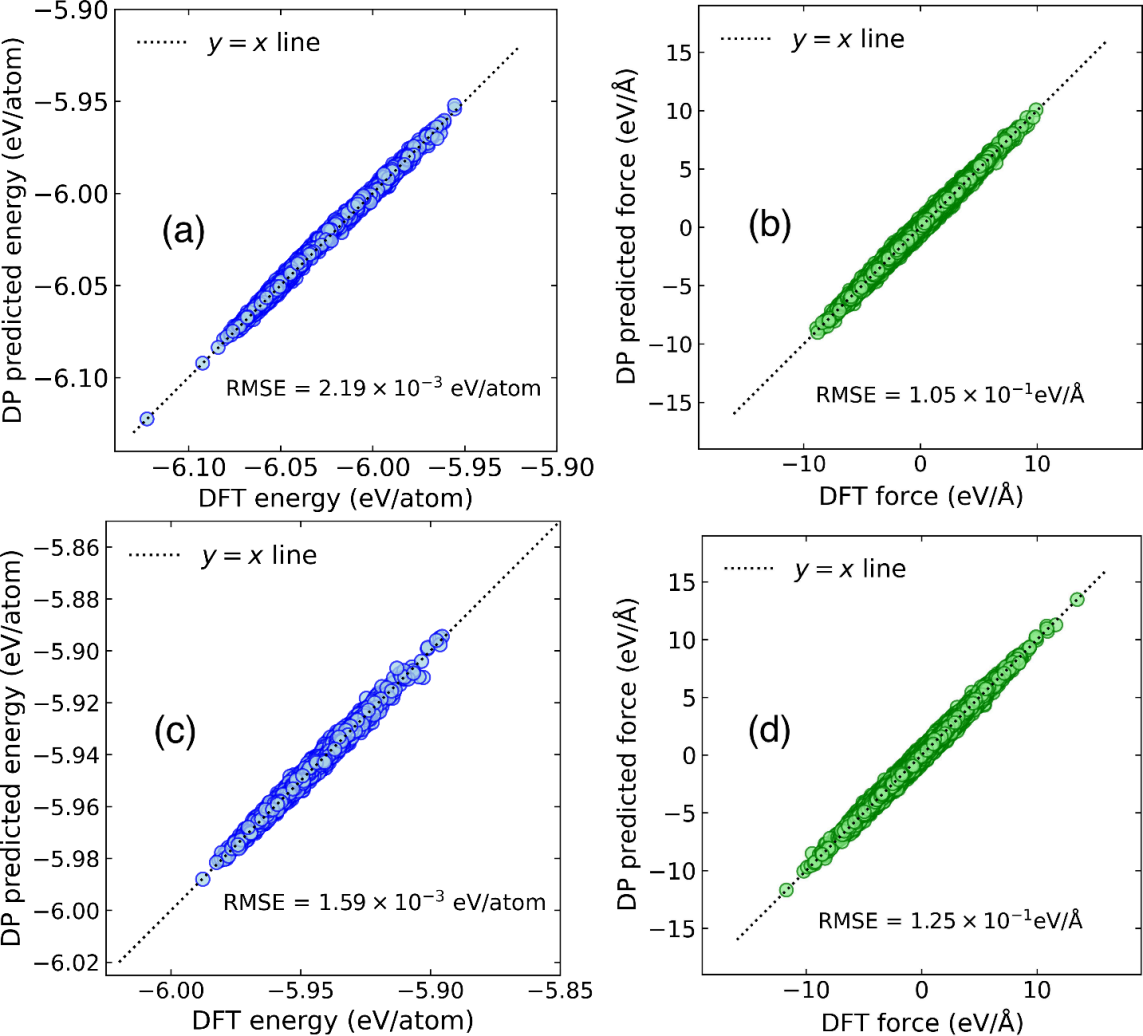
Parity plots for DP-predicted (a) energies and (b) forces for nonprotonated graphamine compared with values computed from DFT data not included in the training data. Parity plot of DP-predicted (c) energies and (d) forces for protonated graphamine compared with values computed from DFT data not included in the training data.

We have verified that our DPs trained on high temperature data accurately reproduce the DFT energies at 300 K by computing parity plots for the p24C system using DFT-MD data not included in the training. These parity plots are presented in . The RMSE errors in energy and forces are 7.2 × 10^–4^ eV/atom and 7.1 × 10^–2^ eV/Å, respectively. These errors are lower than those for the high temperature data shown in Figure 3.

As an additional test of the accuracy of the DPs, we compared the velocity autocorrelation function and phonon density of states for nonprotonated graphamine computed from the DP with calculations from DFT. The DP was not trained on these properties, so these are pure predictions.

#### Velocity Autocorrelation Function

We examined the dynamic properties of graphamine computed from DFT and DP by comparing the velocity autocorrelation functions (VACFs) calculated from *NVT*-MD simulations at *T* = 1200 K for a graphamine system containing 72 atoms from DFT and the trained DP. These systems were first thermally equilibrated for 1.5 ps and then the VACF was calculated using 0.5 ps of an *NVT* production run. We chose 1200 K, the highest temperature for which our DP was trained, as an extreme tests for the DP predictions. The results are plotted in Figure 4. The predictions from the DP VACF match the results from DFT to a high degree of accuracy. The peak positions and amplitudes predicted by the DP agree well with those of the DFT calculations.

**Figure 4:**
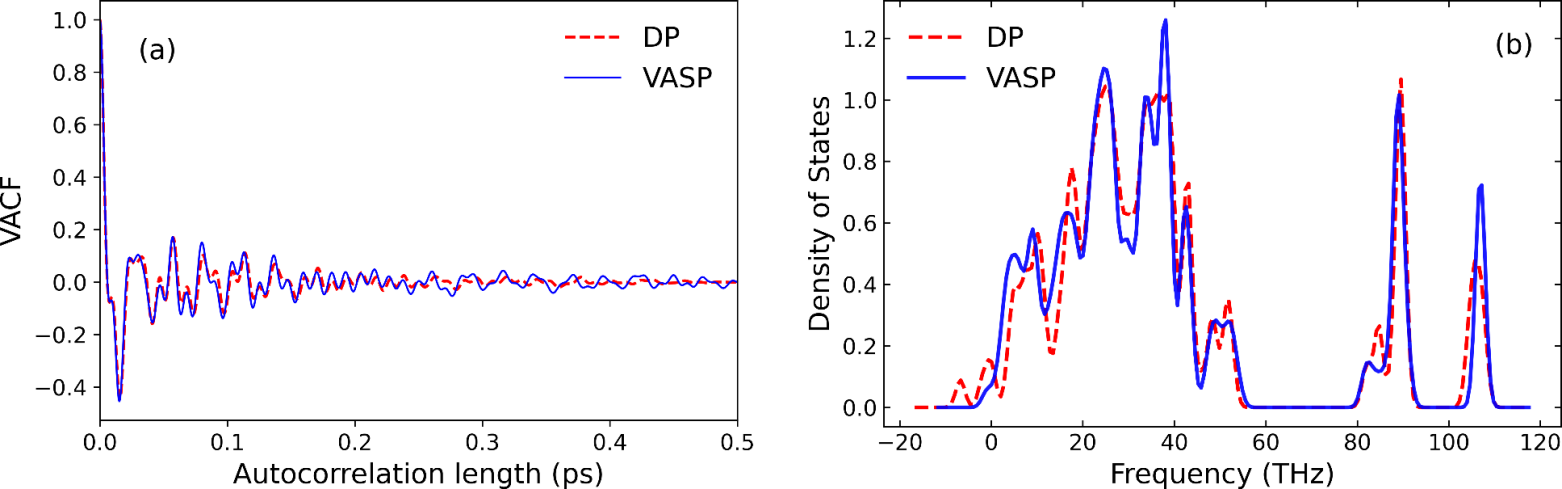
(a) VACF calculated from DFT and DP as a function of autocorrelation length from NVT-MD simulations at *T* = 1200 K and (b) phonon density of states calculated at 0 K from DFT and DP.

#### Phonon Density of States (PDOS)

We computed the PDOS of graphamine using the trained DP and compared it with the corresponding DFT calculations. These results are plotted in Figure 4. Two python tools were used to compute phonon properties: phonopy^52,53^ for DFT-VASP and phonoLAMMPS^54^ for DP-LAMMPS calculations. The phonon calculations were carried out within the quasi-harmonic approximation at 0 K. The PDOS DP predicted is in reasonably good agreement with the DFT calculations. The number of peaks and the peak positions agree very well at positive frequencies. The amplitudes are not in as good agreement. The most significant difference is the appearance of a peak at about −8 THz in the DP PDOS that is absent in the DFT results. In addition, the shoulder in the DFT PDOS at about 0 THz shows up as a distinct peak in the DP PDOS. We plotted the projected DOS from DFT and DP to identify the origin of these differences (see ). We see that the peak at about −8 THz comes from the N atoms and the 4 H atoms that are bound to N atoms (atoms H2, H3, H5, H6 in ). This indicates that the DP could be improved by training to low-frequency modes. However, these modes are unlikely to impact proton transport and we therefore accept the DP without further training.

### Diffusion Coefficients and Proton Conductivity

We computed the proton diffusion coefficient (*D*_*S*_) on graphamine at four different temperatures (300, 350, 400, and 500 K) using LAMMPS *NVT*-MD simulations (see for details). Results are given in Table 1. The values of the proton diffusion coefficient obtained at each temperature were used to estimate the apparent activation energy for diffusion (*E*_*A*_) from an Arrhenius plot, shown in Figure 5. The diffusion coefficients for both graphamine and graphanol (taken from Achar et al.^31^) are compared in Figure 5. The *E*_*A*_ for graphamine is 63 ± 4 meV, whereas the reported *E*_*A*_ for graphanol is 99 ± 13 meV,^31^ which is significantly larger than for graphamine.

**Table 1:** Proton Diffusion Coefficients at Different Temperatures Computed from the DP^a^

*T* (K)	*D*_*S*_ × 10^8^ (m^2^/s)
300	1.21 ± 0.42
350	1.81 ± 0.61
400	2.34 ± 0.87
500	2.68 ± 0.83

a The estimated errors are twice the standard deviations.

**Figure 5:**
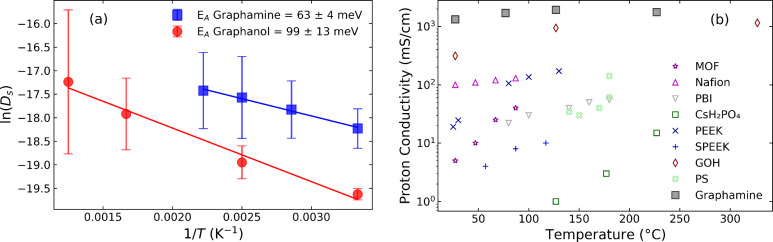
(a) Arrhenius equation fits for graphamine and graphanol.^31^ (b) Proton conductivities for various materials compared with our predictions for graphamine from this work. Data sources: MOF,^55^ Nafion,^56,57^ polybenzimidazole (PBI),^14,58^ solid acids (CsH_2_PO_4_),^59^ poly­(ether ether ketone) (PEEK),^14^ sulfonated poly­(ether ether ketone) (SPEEK),^60^ graphanol (GOH),^31^ and polysulfone (PS).^61,62^

The estimated *E*_*A*_ for graphamine is very low compared to those for other types of materials such as graphanol,^31^ Nafion,^57^ nonmetal–organic frameworks,^22^ and polymeric membranes.^63^ We estimated the proton conductivity of graphamine and compared it with other materials as shown in Figure 5b. We estimated the proton conductivity of graphamine as described in the . We found that the conductivity varies between 1322 and 1757 mS/cm over a temperature range of 300–500 K (27–227 °C). Graphamine exhibits higher diffusivity and conductivity over all temperatures compared to graphanol and other materials. We note that except for graphanol (GOH) in Figure 5b, all other values are experimental, whereas the values for graphamine from this work are theoretical predictions.

### Proton Hopping Barrier

We used our DP to estimate the intrinsic barrier for a single proton hop, *E*_hop_, on graphamine using the climbing image nudged elastic band^64^ method, as described in the . The calculated minimum energy pathway is plotted in Figure 6 and the corresponding initial, transition, and final states are shown in . We calculated a value of *E*_hop_ = 17 meV (average of forward and reverse barriers). This value is somewhat larger than the reported intrinsic activation energy for a proton hop on graphanol of 13 meV.^31^ We note that the intrinsic hopping barrier is significantly lower than the apparent diffusion barrier of 63 meV estimated from the Arrhenius plot in Figure 5. This is because an intrinsic hopping event is most likely to lead to proton rattling rather than long-range proton transport, for which the mechanism is discussed below. We calculated DFT single-point energies for all the images on the computed minimum energy pathway. The DP and DFT relative energies are plotted in Figure 6. We see reasonable agreement between the DP and DFT calculations, with the DP overestimating the barrier by about 3 meV.

**Figure 6:**
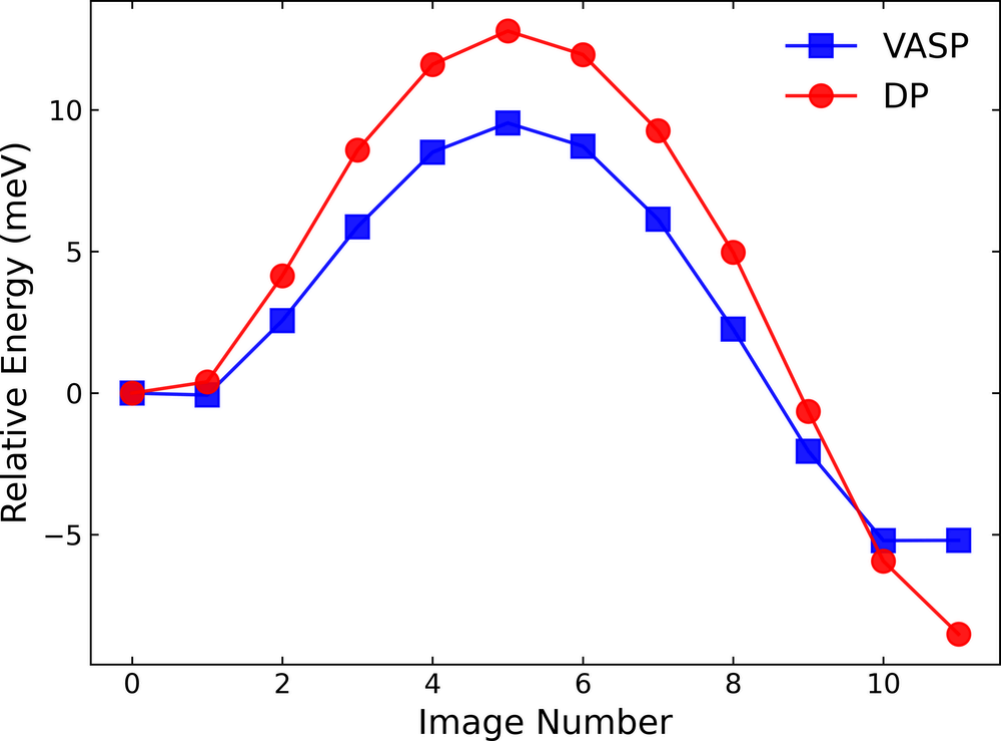
Minimum potential energy surface for a single proton hopping event computed from the DP using the climbing image nudged elastic band method.^64^ Single-point energies computed from DFT are also shown for comparison.

### Proton Transport Mechanism

We observed that proton transport takes place as protons move along 2-D Grotthuss chains, which are formed by hydrogen bonding between neighboring amine groups in graphamine. This is similar to the mechanism observed for proton transport in graphanol.^31^ The proton-bearing NH_3_ group allows a proton to move in one of three directions, corresponding to the three possible hydrogen bonds between the H atoms of NH_3_ and the neighboring N atoms. This is because each of the H atoms on the NH_3_ group may act as the proton. The proton on an NH_3_ group will hop to one of the six neighboring NH_2_ groups. The initial proton will either hop back to the original NH_3_ group (rattling event) or will facilitate a proton hop onto different NH_2_ group. Achar et al.^31^ showed that the activation energy required for proton hopping in graphanol is a convolution of the intrinsic activation energy for proton hopping and the rotational activation energy. This is because long-range proton transport on graphanol requires the formation of new Grotthuss chains by rotation of one or more of the OH groups.^31^ The rotational activation energy for graphanol was reported to be 293 meV.^31^ We expect this value to be higher for graphamine due to the larger number of hydrogen bonds, leading to more hindered rotation in graphamine compared with graphanol. We attempted to compute the rotational barrier for NH_2_ groups on graphamine by computing the rotational diffusion coefficient as a function of temperature, as was done for graphanol.^31^ However, the rotations were too slow to give meaningful results over the time-scale of the simulations (see ), thus confirming our hypothesis that the rotational barrier for NH_2_ rotation is significantly higher than for OH groups on graphanol. Given the larger values of the intrinsic hopping and rotational barriers, one might therefore expect *D*_*S*_ to be lower for graphamine than graphanol, but this is the opposite of what is observed. It therefore stands to reason that the proton transport mechanisms on these two materials are somewhat different. To understand the mechanistic differences of graphamine and graphanol, we constructed spider plots showing the positions of the N or O atoms involved in proton transport for both graphamine and graphanol at the same conditions. These plots are presented in Figure 7. We can see that the plots in Figure 7a are more branched compared to Figure 7b. This is because a proton can hop in three directions on graphamine, compared with two on graphanol. We analyzed the spider plots by calculating the number of nodes (N or O atoms) having *n*-edges, where *n* = 1, ..., 6. The results are plotted in Figure 8, from which we see that graphamine has larger numbers of 3-edged, 4-edged, 5-edged and 6-edged nodes compared to graphanol. The larger number of nodes having 3–6 edges for graphamine compared with graphanol is a direct result of the larger number of directions that a proton can travel on graphamine without significant rotation. Plots identifying the specific nodes having *n* edges for *n* = 3, ..., 6 are given in for graphamine and graphanol, respectively.

**Figure 7:**
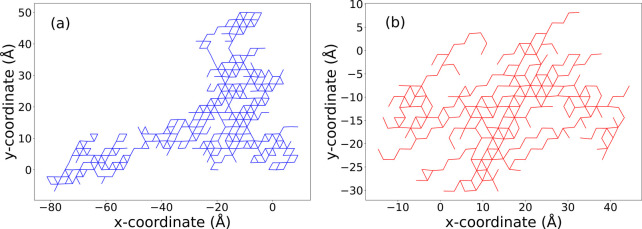
Spider plots comparing proton transport mechanisms in (a) graphamine and (b) graphanol over the same simulation time (125 ps) and at the same temperature (600 K).

**Figure 8:**
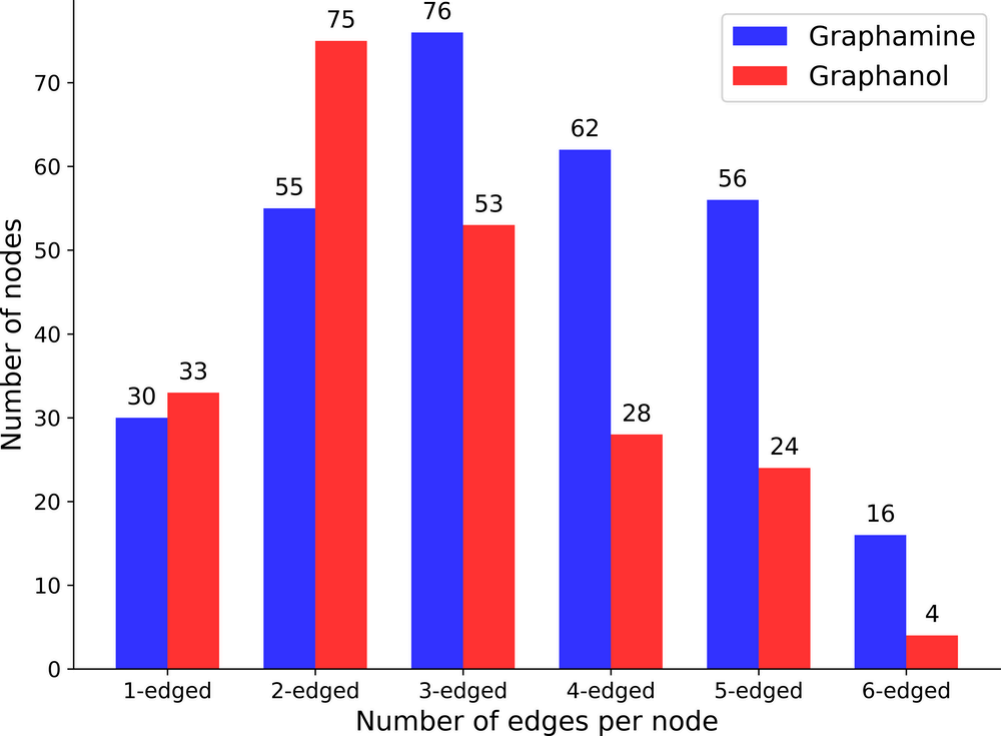
Bar plot containing node-edge analysis of the spider plots shown in Figure 7 for graphamine and graphanol.

We also observed that the length of the Grotthuss chains in graphamine is longer than that of graphanol by observing an animation showing the movement of the CEC, showing the dynamic nature of the Grotthuss chains formed in graphamine and graphanol. The animation is provided in the . The animation shows the dynamic positions of nitrogen or oxygen atoms associated with the CEC, as a function of time. Nodes that have been visited within the previous 0.1 ps are shown as filled circles, which then disappear if a node has not been visited after 0.1 ps. They also depict the history of all CEC positions over the 125 ps simulation, shown as a spider plot. The simulations are for p24C systems at 600 K. The trajectory traced out for graphamine is shown in light blue whereas for graphanol is shown in light red.

As noted above, there is a large discrepancy between the estimated minimum energy pathway barrier for a single proton hop, *E*_hop_ = 17 meV, and the apparent activation energy for diffusion *E*_*A*_ = 63 meV. Note that *E*_hop_ was computed at 0 K and does not include zero point energy corrections (see the ), whereas *E*_*A*_ was computed from statistical mechanics and therefore includes all entropic and vibrational contributions. We have shown that complete rotation of NH_2_ groups is not required for proton diffusion on graphamine. However, we do observe significant librational motion of NH_2_ groups, as seen in . We therefore conclude that *E*_*A*_ is a convolution of *E*_hop_ and the NH_2_ librational energy. This is a reasonable assumption because hopping along a Grotthuss chain for long distances will occur only when several NH_2_ groups in the neighborhood of the NH_3_ moiety are precisely aligned. Thus, the librational energy is very likely the main contributor to the apparent activation energy of diffusion.

## Conclusion

We have constructed an accurate DP and performed atomistic simulations that indicate that graphamine is a promising PEM material for facilitating proton conductivity under anhydrous and low-humidity conditions. We computed proton diffusion coefficients, diffusion activation energy, and proton conductivities for graphamine. We have shown that anhydrous proton conduction on graphamine is possible with an activation energy of 63 meV, which is lower than other membrane materials, such as Nafion,^57^ PBI,^58^ PS,^61^ PI,^14^ PES,^65^ and graphanol,^31^ studied in the literature. Our work provides motivation for the synthesis and characterization of graphane or graphene that has been functionalized with amine groups. We note that while our system is fully functionalized on one side, it is desirable to have a material that is functionalized on both sides, since this would increase the conductivity.

We discussed the details of the proton hopping mechanism and compared it with that of graphanol. We presented spider plots that show that protons hop along Grotthuss chains, which are pathways of hydrogen bonds between neighboring amine groups allowing proton conduction. For graphanol, long-range proton transport requires formation of new Grotthuss chains through OH group rotations. In contrast, we have shown full rotation of NH_2_ groups on graphamine is not required to facilitate proton diffusion. Instead, only librational motion of the NH_2_ groups is needed to achieve proton diffusion to all possible sites. This is because of the higher degree of connectivity in graphamine, since each NH_2_ group can participate in four hydrogen bonds, two donors and two acceptors. In graphanol each OH group can only form two hydrogen bonds, one donor and one acceptor. We believe it is this feature that gives a lower proton diffusion activation energy compared with graphanol.

There are several limitations to our calculations that could impact the accuracy of our predictions compared with proton diffusion on experimentally produced graphamine. We have used a generalized gradient approximation exchange-correlation functional in our DFT calculations. This class of functionals is known to suffer from self-interaction errors that typically reduce reaction barriers.^66^ Indeed, these functionals have been shown to underestimate proton hopping barriers in water compared with accurate wave function methods.^67^ Our computed barriers and diffusion coefficients may therefore be too low and too high, respectively. We have also ignored nuclear quantum effects, which act to decrease the barriers and increase the diffusivity.^29^ Thus, there is some degree of error cancellation in our results. Another shortcoming is that real graphamine will contain defects, such as missing NH_2_ groups due to incomplete functionalization. Importantly, complete functionalization is not required for effective proton transport, since the percolation threshold for a triangular lattice is 0.5.^31^ The impact of defects on proton diffusion is beyond the scope of this work, but could be addressed in a future publication. Notwithstanding these limitations, we believe we have shown that experimental work to measure proton conduction on graphamine is warranted.

## Supplementary Material





## Data Availability

The codes for estimating diffusivity using CEC and proton transport mechanism analysis are publicly available at https://github.com/Lakshmi-Ananthabhotla/proton_conduction_graphamine.

## Notes

Atomic coordinates for graphamine, details of the DeePMD training architecture, the DP active learning procedure, the center of excess charge calculations, phonon density of states calculations, bond dissociation energy calculations, proton conductivity calculations, the mean squared displacement calculations and plots, the intrinsic proton hopping barrier, amine group rotations, and proton transport mechanism (PDF)
Animations showing the dynamics of proton hopping on graphamine and graphanol. (MP4)

## ¶

Pritzker School of Molecular Engineering, University of Chicago, Chicago, Illinois 60637, United States

## References

[1] Wee J.-H. (2007). Applications
of proton exchange membrane fuel cell systems. Renewable Sustainable Energy Rev..

[2] Carrette L., Friedrich K. A., Stimming U. (2000). Fuel cells: principles, types, fuels,
and applications. ChemPhysChem.

[3] Kreuer K.-D. (1996). Proton
conductivity: materials and applications. Chem.
Mater..

[4] Brunello G., Lee S. G., Jang S. S., Qi Y. (2009). A molecular dynamics
simulation study of hydrated sulfonated poly (ether ether ketone)
for application to polymer electrolyte membrane fuel cells: Effect
of water content. J. Renewable Sustainable Energy.

[5] Brandell D., Karo J., Liivat A., Thomas J. O. (2007). Molecular dynamics
studies of the Nafion®, Dow® and Aciplex® fuel-cell
polymer membrane systems. J. Mol. Model..

[6] Alberti G., Casciola M., Massinelli L., Bauer B. (2001). Polymeric proton conducting
membranes for medium temperature fuel cells (110–160 C). J. Membr. Sci..

[7] Komarov P. V., Khalatur P. G., Khokhlov A. R. (2013). Large-scale atomistic
and quantum-mechanical
simulations of a Nafion membrane: Morphology, proton solvation and
charge transport. Beilstein J. Nanotechnol..

[8] Hwang G. S., Kaviany M., Gostick J. T., Kientiz B., Weber A. Z., Kim M. H. (2011). Role of water states
on water uptake and proton transport
in Nafion using molecular simulations and bimodal network. Polymer.

[9] Alaswad A., Omran A., Sodre J. R., Wilberforce T., Pignatelli G., Dassisti M., Baroutaji A., Olabi A. G. (2021). Technical and commercial challenges of proton-exchange
membrane (PEM). fuel cells. Energies (Basel,
Switz.).

[10] Xiao T., Wang R., Chang Z., Fang Z., Zhu Z., Xu C. (2020). Electrolyte membranes
for intermediate temperature proton exchange
membrane fuel cell. Prog. Nat. Sci.:Mater. Int..

[11] Li Q., He R., Jensen J. O., Bjerrum N. J. (2003). Approaches and Recent Development
of Polymer Electrolyte Membranes for Fuel Cells Operating above 100°C. Chem. Mater..

[12] Nagarkar S. S., Unni S. M., Sharma A., Kurungot S., Ghosh S. K. (2014). Two-in-one:
inherent anhydrous and water-assisted high proton conduction in a
3D metal-organic framework. Angew. Chem., Int.
Ed..

[13] Schuster M. F., Meyer W. H. (2003). Anhydrous proton-conducting polymers. Annu. Rev. Mater. Res..

[14] Karimi M. B., Hooshyari K., Salarizadeh P., Beydaghi H., Ortiz- Martinez V.M., Ortiz A., Ortiz Uribe I., Mohammadi F. (2021). A comprehensive
review on the proton conductivity of proton exchange membranes (PEMs)
under anhydrous conditions: Proton conductivity upper bound. Int. J. Hydrogen Energy.

[15] Paddison S. (2003). Proton conduction
mechanisms at low degrees of hydration in sulfonic acid-based polymer
electrolyte membranes. Annu. Rev. Mater. Res..

[16] Peighambardoust S. J., Rowshanzamir S., Amjadi M. (2010). Review of the proton exchange membranes
for fuel cell applications. Int. J. Hydrogen
Energy.

[17] Klumpen C., Winterstein S., Papastavrou G., Senker J. (2018). Anhydrous proton conduction
in porous organic networks. J. Mater. Chem.
A.

[18] Hurd J. A., Vaidhyanathan R., Thangadurai V., Ratcliffe C. I., Moudrakovski I. L., Shimizu G. K. (2009). Anhydrous proton conduction at 150
C in a crystalline metal-organic framework. Nat. Chem..

[19] Chandra S., Kundu T., Kandambeth S., BabaRao R., Marathe Y., Kunjir S. M., Banerjee R. (2014). Phosphoric
Acid Loaded Azo (-N =
N-) Based Covalent Organic Framework for Proton Conduction. J. Am. Chem. Soc..

[20] Peng Y., Xu G., Hu Z., Cheng Y., Chi C., Yuan D., Cheng H., Zhao D. (2016). Mechanoassisted synthesis of sulfonated
covalent organic frameworks with high intrinsic proton conductivity. ACS Appl. Mater. Interfaces.

[21] Xu H., Tao S., Jiang D. (2016). Proton conduction
in crystalline and porous covalent
organic frameworks. Nat. Mater..

[22] O’Shaughnessy M., Lim J., Glover J., Neale A. R., Day G. M., Hardwick L. J., Cooper A. I. (2025). Nonmetal Organic Frameworks Exhibit High Proton Conductivity. J. Am. Chem. Soc..

[23] Chakraborty C., Rana U., Pandey R. K., Moriyama S., Higuchi M. (2017). One-dimensional
anhydrous proton conducting channel formation at high temperature
in a pt (ii)-based metallo-supramolecular polymer and imidazole system. ACS Appl. Mater. Interfaces.

[24] Pawlaczyk C., Pawłowski A., Połomska M., Pogorzelec-Glaser K., Hilczer B., Pietraszko A., Markiewicz E., Ławniczak P., Szcześniak L. (2010). Anhydrous
proton conductors for use
as solid electrolytes. Phase Transitions.

[25] Ramaswamy P., Wong N. E., Shimizu G. K. (2014). MOFs as
proton conductors-challenges
and opportunities. Chem. Soc. Rev..

[26] Mukherjee D., Saha A., Moni S., Volkmer D., Das M. C. (2025). Anhydrous
Solid-State Proton Conduction in Crystalline MOFs, COFs, HOFs, and
POMs. J. Am. Chem. Soc..

[27] Bagusetty A., Livingston J., Johnson J. K. (2019). Graphamine: Amine-Functionalized
Graphane for Intrinsic Anhydrous Proton Conduction. J. Phys. Chem. C.

[28] Wang H., Zhang L., Han J., Weinan E. (2018). DeePMD-kit:
A deep
learning package for many-body potential energy representation and
molecular dynamics. Comput. Phys. Commun..

[29] Bagusetty A., Choudhury P., Saidi W. A., Derksen B., Gatto E., Johnson J. K. (2017). Facile
anhydrous proton transport on hydroxyl functionalized
graphane. Phys. Rev. Lett..

[30] Bagusetty A., Johnson J. K. (2019). Unraveling anhydrous
proton conduction in hydroxygraphane. J. Phys.
Chem. Lett..

[31] Achar S. K., Bernasconi L., DeMaio R. I., Howard K. R., Johnson J. K. (2023). In silico
demonstration of fast anhydrous proton conduction on graphanol. ACS Appl. Mater. Interfaces.

[32] Achar S. K., Bernasconi L., Alvarez J. J., Johnson J. K. (2023). Deep-learning potentials
for proton transport in double-sided graphanol. J. Mater. Res..

[33] Stepanova M., Solomakha O., Rabchinskii M., Averianov I., Gofman I., Nashchekina Y., Antonov G., Smirnov A., Ber B., Nashchekin A. (2021). Aminated graphene-graft-oligo (glutamic
acid)/poly (*ε*-caprolactone) composites: Preparation,
characterization and biological evaluation. Polymers (Basel, Switz.).

[34] Rabchinskii M. K., Ryzhkov S. A., Kirilenko D. A., Ulin N. V., Baidakova M. V., Shnitov V. V., Pavlov S. I., Chumakov R. G., Stolyarova D. Y., Besedina N. A. (2020). From graphene oxide towards aminated graphene:
Facile synthesis, its structure and electronic properties. Sci. Rep..

[35] Wang B., Luo B., Liang M., Wang A., Wang J., Fang Y., Chang Y., Zhi L. (2011). Chemical amination of graphene oxides
and their extraordinary properties in the detection of lead ions. Nanoscale.

[36] Kresse G., Hafner J. (1993). Ab initio molecular dynamics for liquid metals. Phys. Rev. B.

[37] Kresse G., Hafner J. (1994). Ab initio molecular-dynamics
simulation of the liquid-metal-amorphous-semiconductor
transition in germanium. Phys. Rev. B.

[38] Kresse G., Furthmüller J. (1996). Efficient
iterative schemes for ab initio total-energy
calculations using a plane-wave basis set. Phys.
Rev. B.

[39] Kresse G., Furthmüller J. (1996). Efficiency
of ab-initio total energy calculations for
metals and semiconductors using a plane-wave basis set. Comput. Mater. Sci..

[40] Blöchl P. E. (1994). Projector
augmented-wave method. Phys. Rev. B.

[41] Hammer B., Hansen L. B., Nørskov J. K. (1999). Improved
adsorption energetics within
density-functional theory using revised Perdew-Burke-Ernzerhof functionals. Phys. Rev. B.

[42] Zhang Y., Yang W. (1998). Comment on “Generalized
gradient approximation made simple”. Phys. Rev. Lett..

[43] Grimme S., Ehrlich S., Goerigk L. (2011). Effect of
the damping function in
dispersion corrected density functional theory. J. Comput. Chem..

[44] Jing A., Szalewicz K., van der Avoird A. (2022). Ammonia dimer: extremely fluxional
but still hydrogen bonded. Nat. Commun..

[45] Neese F. (2012). The ORCA program
system. WIRES Comput. Mol. Sci..

[46] Evans D. J., Holian B. L. (1985). The nose-hoover
thermostat. J.
Chem. Phys..

[47] Zeng J., Zhang D., Lu D., Mo P., Li Z., Chen Y., Rynik M., Huang L., Li Z., Shi S. (2023). DeePMD-kit v2: A software package for deep potential
models. J. Chem. Phys..

[48] Achar S. K., Zhang L., Johnson J. K. (2021). Efficiently Trained Deep Learning
Potential for Graphane. J. Phys. Chem. C.

[49] Zhang Y., Wang H., Chen W., Zeng J., Zhang L., Wang H. (2020). DP-GEN:
A concurrent learning platform for the generation
of reliable deep learning based potential energy models. Comput. Phys. Commun..

[50] Thompson A. P., Aktulga H. M., Berger R., Bolintineanu D. S., Brown W. M., Crozier P. S., in ’t Veld P. J., Kohlmeyer A., Moore S. G., Nguyen T. D. (2022). LAMMPS
- a flexible simulation tool for particle-based materials modeling
at the atomic, meso, and continuum scales. Comput.
Phys. Commun..

[51] Li C., Swanson J. M. (2020). Understanding and tracking the excess proton in Ab
Initio simulations; Insights from IR spectra. J. Phys. Chem. B.

[52] Togo A., Chaput L., Tadano T., Tanaka I. (2023). Implementation
strategies
in phonopy and phono3py. J. Phys.: Condens.
Matter.

[53] Togo A. (2023). First-principles
Phonon Calculations with Phonopy and Phono3py. J. Phys. Soc. Jpn..

[54] Carreras, A. LAMMPS interface for phonon calculations using phonopy. https://github.com/abelcarreras/phonolammps, 2019.

[55] Sun X., Simonsen S. C., Norby T., Chatzitakis A. (2019). Composite
membranes for high temperature PEM fuel cells and electrolysers: a
critical review. Membranes (Basel, Switz.).

[56] Cappadonia M., Erning J. W., Niaki S. M. S., Stimming U. (1995). Conductance of Nafion
117 membranes as a function of temperature and water content. Solid State Ionics.

[57] Feng S., Voth G. A. (2011). Proton solvation and transport in
hydrated nafion. J. Phys. Chem. B.

[58] Hu J., Luo J., Wagner P., Conrad O., Agert C. (2009). Anhydrous proton conducting
membranes based on electron-deficient nanoparticles/PBI-OO/PFSA composites
for high-temperature PEMFC. Electrochem. Commun..

[59] Goñi-Urtiaga A., Presvytes D., Scott K. (2012). Solid acids as electrolyte materials
for proton exchange membrane (PEM) electrolysis. Int. J. Hydrogen Energy.

[60] Mayahi A., Ismail A., Ilbeygi H., Othman M., Ghasemi M., Norddin M. M., Matsuura T. (2013). Effect of
operating temperature on
the behavior of promising SPEEK/cSMM electrolyte membrane for DMFCs. Sep. Purif. Technol..

[61] Bai H., Wang H., Zhang J., Zhang J., Lu S., Xiang Y. (2019). High temperature polymer electrolyte membrane achieved by grafting
poly (1-vinylimidazole) on polysulfone for fuel cells application. J. Membr. Sci..

[62] Li J., Wu H., Cao L., He X., Shi B., Li Y., Xu M., Jiang Z. (2019). Enhanced proton conductivity of sulfonated
polysulfone
membranes under low humidity via the incorporation of multifunctional
graphene oxide. ACS Appl. Nano Mater..

[63] Narayanan S., Yen S.-P., Liu L., Greenbaum S. (2006). Anhydrous
proton-conducting polymeric electrolytes for fuel cells. J. Phys. Chem. B.

[64] Henkelman G., Uberuaga B. P., Jónsson H. (2000). A climbing image nudged elastic band
method for finding saddle points and minimum energy paths. J. Chem. Phys..

[65] Zhang J., Lu S., Zhu H., Chen K., Xiang Y., Liu J., Forsyth M., Jiang S. P. (2016). Amino-functionalized mesoporous silica
based polyethersulfone-polyvinylpyrrolidone composite membranes for
elevated temperature proton exchange membrane fuel cells. RSC Adv..

[66] Shukla P. B., Mishra P., Baruah T., Zope R. R., Jackson K. A., Johnson J. K. (2023). How Do Self-Interaction Errors Associated with Stretched
Bonds Affect Barrier Height Predictions?. J.
Phys. Chem. A.

[67] Sadhukhan S., Muñoz D., Adamo C., Scuseria G. E. (1999). Predicting
proton
transfer barriers with density functional methods. Chem. Phys. Lett..

