# Supplementary material for: Proton Transport on Graphamine: A Deep-Learning Potential Study
Source: J Phys Chem C Nanomater Interfaces. 2025 Nov 14;129(47):20880–8. doi: 10.1021/acs.jpcc.5c05356 (PMC12670509; doi:10.1021/acs.jpcc.5c05356)
Supplement: Supplementary file 1 [file jp5c05356_si_001.pdf]

Supporting Information: Proton Transport on Graphamine: A Deep-Learning Potential Study

Lakshmi Y. Ananthabhotla,[†] Siddarth K. Achar,[‡,¶] and J. Karl Johnson[*,†]

†*Department of Chemical & Petroleum Engineering, University of Pittsburgh, Pittsburgh, PA, 15261, USA*

‡*Computational Modeling & Simulation Program, University of Pittsburgh, Pittsburgh, PA, 15260, USA*

¶*Current address: Pritzker School of Molecular Engineering, University of Chicago, Chicago, Illinois 60637, United States*

E-mail: karlj@pitt.edu

Atomic Coordinates

Fractional coordinates for the p24C graphamine unit cell.

```
    GNH2
     1.0000000000000000
     10.4774036914797293    0.0000000000000000    0.0000000000000000
    -3.9290263843049007    6.8052733218947283    0.0000000000000000
     0.0000000000000000    0.0000000000000000   20.0000000000000000
 C   N   H
  24  12  37
Direct
  0.3753960213286546  0.6731188079838509  0.1253635767532432
  0.2928763216216639  0.4517616742812604  0.1006137367618633
  0.1252973401001011  0.0053906943876009  0.1228528231571244
  0.5423785187402650  0.7833517749945450  0.1018597355317636
  0.2922302642616155  0.1168981124160138  0.0996274446060451
  0.3753783839569453  0.3385298393626865  0.1249083618430970
  0.6246102678947835  0.6715400673748465  0.1264173587599167
  0.5423799342632197  0.4496743406578888  0.1017338791210954
  0.3758550321678585  0.0058774144610965  0.1244716461222216
  0.7908866040280351  0.7825456906498763  0.1012470614676628
  0.6261212759133395  0.3393571886722057  0.1262038093289086
  0.5434402087146061  0.1186074133311094  0.1009569716953364
  0.8755983505904816  0.6726630185019489  0.1250130426424887
  0.7928863816592413  0.4499456435836803  0.1016792899459537
  0.6262185916037416  0.0065534141480012  0.1254955247719277
  0.8773066133839886  0.3400315764118531  0.1257974178281209
```

0.7926281750526636	0.1170570025422747	0.1019177250760482
0.8746983539310214	0.0051746682732126	0.1256273024073281
0.0419254905136850	0.1172342932066526	0.1008745925007001
0.1263579589023751	0.3397643549964379	0.1249227772575456
0.2931846524403976	0.7849582480066312	0.1001922075905798
0.0426028375666421	0.4499167800894863	0.1002034050936643
0.1257608403647965	0.6730797485879889	0.1236442976040365
0.0409875222820913	0.7829802022832945	0.0995919904739819
0.3645230918567737	0.6697570887905577	0.1992724152401176
0.1276209858973208	0.9964248633524481	0.1979202945905022
0.3704139519430285	0.3301472641302842	0.1991786536577623
0.6167228551998704	0.6670810706722613	0.2009551028506046
0.3819835314377744	0.0119548558655599	0.1985807655850313
0.6313238342555731	0.3444914118813519	0.2008780281824013
0.6229584942255861	0.0039492931102085	0.1997443394385073
0.8756919507431736	0.6791548646385845	0.1994333972468313
0.8857342678610975	0.3523066328531156	0.1999054621503579
0.8752871369808241	0.0046786004184144	0.2000806590982908
0.1285821930040632	0.3268422487207537	0.1985687483384332
0.1262808644497714	0.6758086689425973	0.1980963309811624
0.0393133810026815	0.9969244325049539	0.2185859900836217
0.2705905254108503	0.3327419527511433	0.2107103493510940
0.2936255230701010	0.4543176210758677	0.0455771601453030
0.4480759535247429	0.7907489331810544	0.2200159088905828
0.3722507276716432	0.5571089882816318	0.2181537726697162
0.1293530409517706	0.8587539040672161	0.2091317667202764
0.2211796573009102	0.1160881604776007	0.2158110015881063

0.2923540013473642	0.1173173281487898	0.0446002490229549
0.5429106653446124	0.7826656886728108	0.0468003406784334
0.4632401917264545	0.4551356076755059	0.2162590016772915
0.6084279043442519	0.7869842136738114	0.2154724066438760
0.2953735129460187	0.8951830580655155	0.2197600379287254
0.3797861073649619	0.1398816727940289	0.2117698094860908
0.5436231677834982	0.4505902177556214	0.0466742391227879
0.5520470744079538	0.2163868974119108	0.2202689758602658
0.7144146167591191	0.6935110454094497	0.2198853624589060
0.7887382951763457	0.7810514901624284	0.0461939778446226
0.5456445721868804	0.1228574245263524	0.0459330980714152
0.6038533882937572	0.4449758679792862	0.2165229736624673
0.5244211911253965	0.0046891427227459	0.2110760575898380
0.7923766590187805	0.4480161489069494	0.0466467716293352
0.7015998602072713	0.1355792634469507	0.2167039157258005
0.7935137922749577	0.3531070322938046	0.2172911351961017
0.9663303239776493	0.6831110815755087	0.2183596131928766
0.7907136635875496	0.5485257500352551	0.2158127324623492
0.7926613151655468	0.1171568616053321	0.0468347319142771
0.7757077934881399	0.9824237669209775	0.2160038629560931
0.8944159115691814	0.2395263464262665	0.2194024973905472
0.8734300019767972	0.8731587934065950	0.2125381693046998
0.0403722693650704	0.1164657381681362	0.0458609743086918
0.2948532080201484	0.7859379899898605	0.0451606284866601
0.2231610038628805	0.6679137810648230	0.2105513140433782
0.0308951589864384	0.2062462218345922	0.2132177565053855
0.0415638175409034	0.4472818450807272	0.0451248150845083

```
  0.0285399093962883  0.5411894094983264  0.2112484680555171
  0.0385064906109157  0.7817783000910697  0.0446029514647570
  0.1373685005260625  0.4454460093372817  0.2216433848970456
```

Fractional coordinates for unit cell of graphamine used for phonopy calculations.

```
   GNH2
     1.00000000000000
     5.6177312721862176    0.0000000000000000    0.0000000000000000
    -4.0296585353724268    2.1386967044869496    0.0000000000000000
     0.0000000000000000    0.0000000000000000   20.5123875559808049
   C    N    H
     4     2     6
Direct
  0.2483517041135094  0.4020143918041465  0.1033019018315769
  0.2444617588471342  0.0691815917049125  0.1272006394852931
  0.7483265026639242  0.4019924495168722  0.1033039430519688
  0.7444847401770477  0.0691977724917522  0.1272004744234694
  0.2581909927707614  0.0782006387810595  0.1990422228416617
  0.7583858629775851  0.0783625014929614  0.1990419161197799
  0.2458292660225070  0.4022910270058161  0.0495517746570893
  0.9167826397387379  0.7731286077943088  0.2160958596400984
  0.4978643234218071  0.5097391663488968  0.2125840314436686
  0.7457721443108788  0.4022288680087166  0.0495534292944312
  0.4171677580425939  0.7736931686167966  0.2161170001113877
  0.9982271805658967  0.5100382326438923  0.2125664885278797
```

DeePMD Training Architecture

We used the DeePMD framework to train deep-learning potentials (DPs).[S1,S2] An ensemble of four DPs was fitted to the training data consisting of DFT calculations on the periodic cells of graphamine. Each DP takes atomic coordinates as input, which are passed through an embedding neural network and then a fitting neural network to produce total energy and forces as outputs. The total energy of the system is calculated as the sum of individual atomic energies. To describe each atom's environment, a cutoff radius of 6 Å and a smoothing cutoff radius of 2 Å were used to count neighboring atoms within this range. These descriptors are fed into the fitting neural network to estimate the energy of each atom. The training dataset was divided into several batches, each containing a fixed number of samples defined by the batch size parameter, which facilitates efficient and stable training of each DP model.

Active Learning Using DP-GEN

We refined the DPs through active learning using the DP-GEN scheme.[S3] In each iteration, an ensemble of four DPs was trained, and LAMMPS-MD simulations were performed using one randomly selected DP to explore new configurations of the potential energy surface. The remaining three DPs were used to calculate forces and energies for the sampled configurations. Configurations were filtered using the maximum force deviation metric employed in DP-GEN,[S3] defined as

$$\epsilon = \max_i \left[\sqrt{\langle || F_{\omega,i}(\mathcal{R}) - \langle F_{\omega,i}(\mathcal{R}) \rangle ||^2 \rangle} \right], \tag{S1}$$

where $F_{\omega,i}(\mathcal{R})$ is the force on atom i in configuration $\mathcal{R}$, ω denotes the DP parameters, and the angle brackets $\langle \ldots \rangle$ represent the average over the DP ensemble. Configurations with $\epsilon <$ 0.1 eV/Å were deemed reliable, those with $\epsilon > 1$ eV/Å were rejected (deemed too unreliable for relabeling), and intermediate cases were relabeled with DFT. Our convergence criterion

was that if more than 97% of configurations fell below the lower uncertainty threshold, the active learning process was terminated, and the resulting DPs were used to study proton conduction in graphamine. The training converged after eight active learning cycles. All DPs were trained on NVIDIA A100 GPUs with an average wall time of 510 minutes per training, and the average wall time for LAMMPS-MD simulations (25 ps each) was 45 minutes on 1 NVIDIA A100 GPU. Parity plots for the training data are shown in Figure S1.

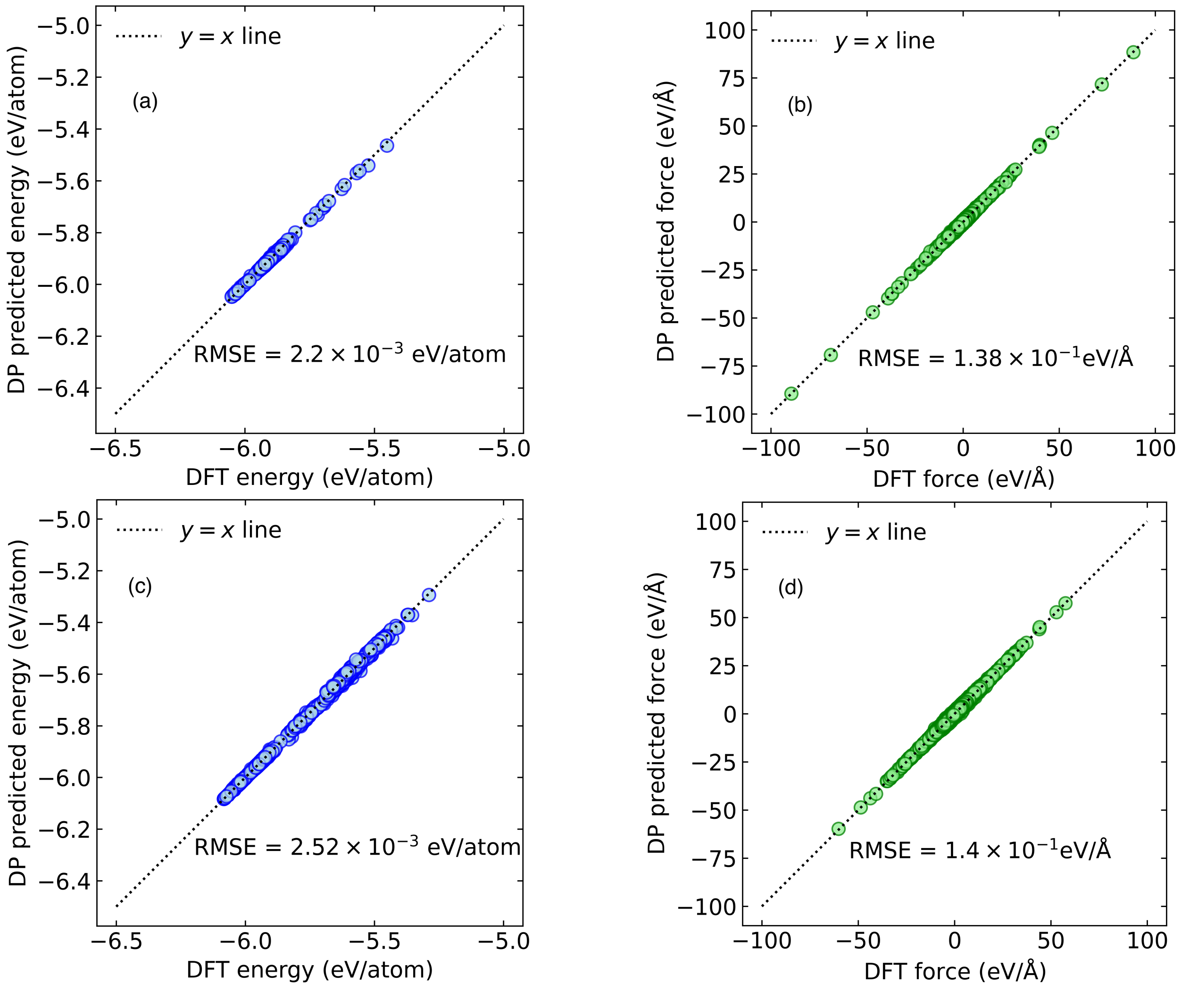

Figure S1: Parity plots of training data for (a) energies and (b) forces for n24C graphamine. Parity plots of training data for (c) energies and (d) forces for p24C graphamine.

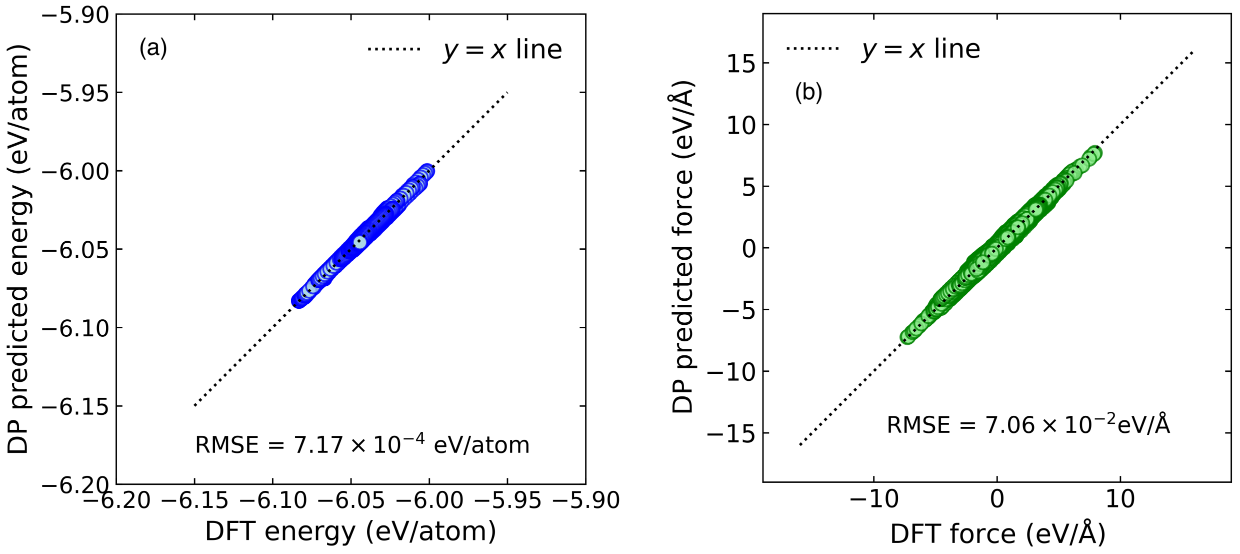

Figure S2: Parity plots for DFT-MD data for the p24C graphamine system at 300 K for (a) energies and (b) forces. These data were not included in the training set.

Center of Excess Charge

The CEC for a given configuration is defined as the position of the N atom that has three H atoms within a cutoff radius of 1.2 Å. Due to fluctuations in N–H bond distances, it is possible for multiple nitrogen atoms to simultaneously satisfy this criterion. In such cases—particularly at time steps where there is no history of the previous CEC position (e.g., the first frame after equilibration)—we assign the CEC to the N atom with the smallest total N–H bond distance, determined by summing the distances to its three nearest H neighbors. The N atom with the smallest sum of N-H distances is picked as the CEC for that time step. An important limitation is that there should only be one CEC at any given time, since there is only a single added proton in the systems we are studying. The main constraint for this methodology required the CEC at time $t + \delta t$ during an MD simulation to be within the first nearest neighbors of the CEC at time t. This means that the CEC can either stay on the original N atom or hop onto one of its six neighboring N atoms over a single time step. This nearest neighbor constraint eliminates the possibility of misassignment of the CEC due to fluctuations in N-H bond distances away from the actual CEC.

Phonon Density of States (PDOS)

The projected phonon density of the states (PDOS) of a unit cell containing 12 atoms was calculated using the DP and compared to the phonopy[S4,S5] calculations using VASP. These PDOS plots are shown in Figure S3. The unit cell used for the PDOS calculations and the identity of the atoms for the partial PDOS plots are given in Figure S4. We also computed the thermodynamic properties from DP and DFT. These are shown in Figure S5 where we plot the Gibbs free energy, entropy and heat capacity. We see that the DP underpredicts the entropy, which causes the Gibbs free energy to be overpredicted.

Bond Dissociation Energies

We calculated the energies required to break a C–H bond and a C–NH_3 bond. We did this by gradually increasing either the C–H bond distance or the C–NH_3 bond distance by 1.5 Å. The initial and final structures are shown in Figure S6. We calculated DFT single-point energies for each of these structures and added them to the training data. A comparison of the energies computed from the trained DP and the DFT calculations are shown in Figure S7. The DP slightly underestimates the dissociation energy for C–NH_3 dissociation, but if this were a significant issue, we would observe NH_3 groups detaching from the surface during our DP-MD simualtions. Since no such events occur, the dissociation energy is sufficiently accurate for our purposes. Our goal is not to reproduce the DFT dissociation energy exactly, but rather to ensure high enough accuracy to avoid spurious dissociation events at the temperatures relevant to our diffusion calculations. This criterion has been successfully met.

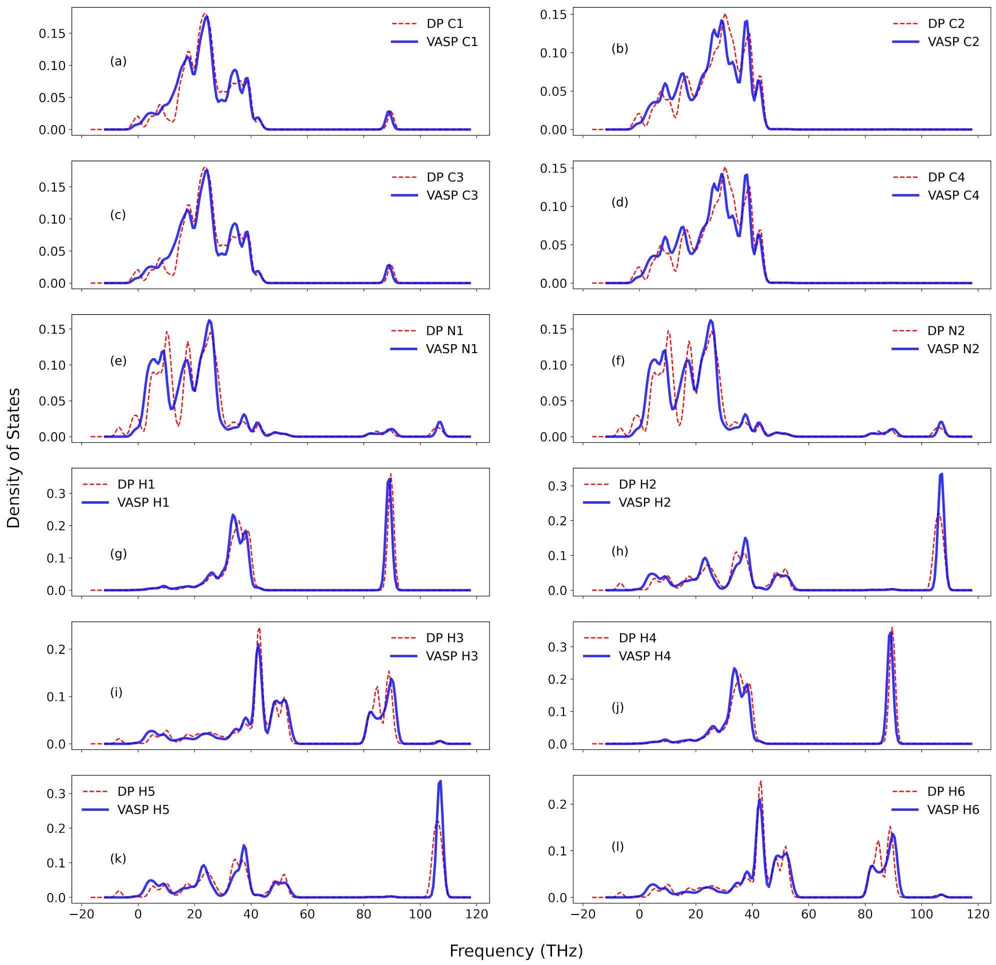

Figure S3: Partial phonon density of states for non-protonated graphamine (n24C) calculated from density functional theory (solid blue line) and from the DP (dashed red line). (a-d) Modes from C atoms, (e-f) modes from N atoms, (g-l) modes from H atoms. The atom labels correspond to those shown in Figure S4.

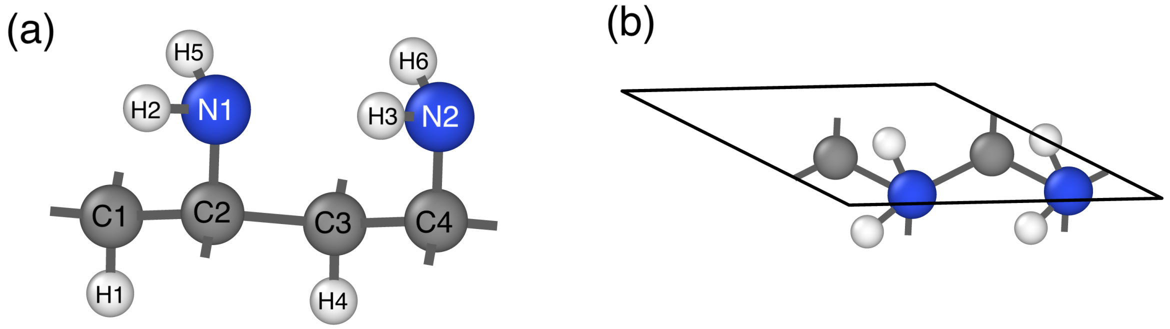

Figure S4: (a) Side-view and (b) top-view of the unit cell of graphamine used for PDOS calculations. The atom labels in this figure correspond to the atom labels used in Figure S3.

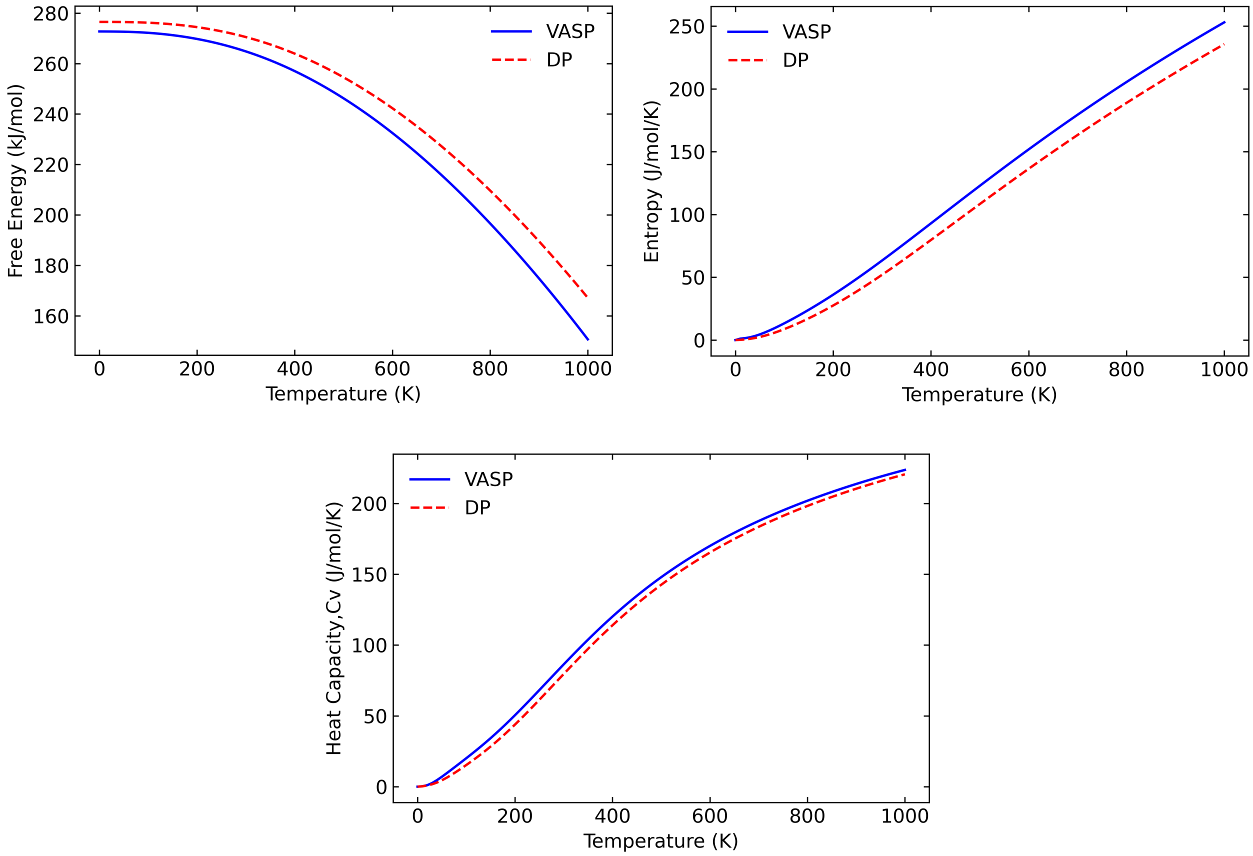

Figure S5: Thermodynamic properties of graphamine estimated using the phonon density of states calculations.

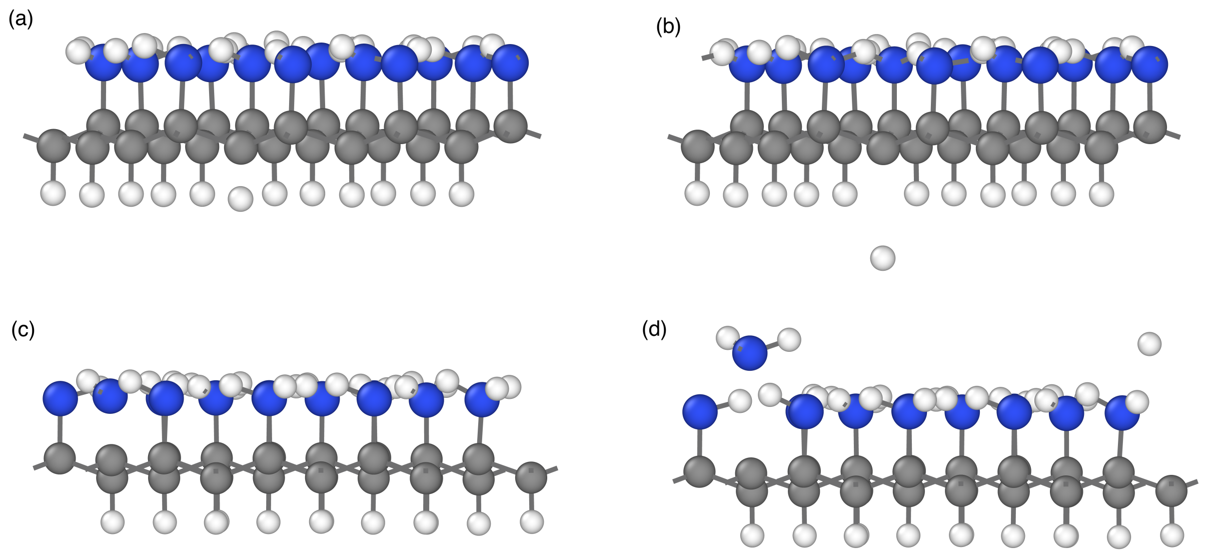

Figure S6: (a) Initial and (b) final configurations used in estimating the C–H bond dissociation energy. (c) Initial and (d) final configurations used in estimating the C–NH_3 bond dissociation energy. Note that one H atom on the NH_3 group appears on the right side of the cell due to periodic boundary conditions in (c) and (d).

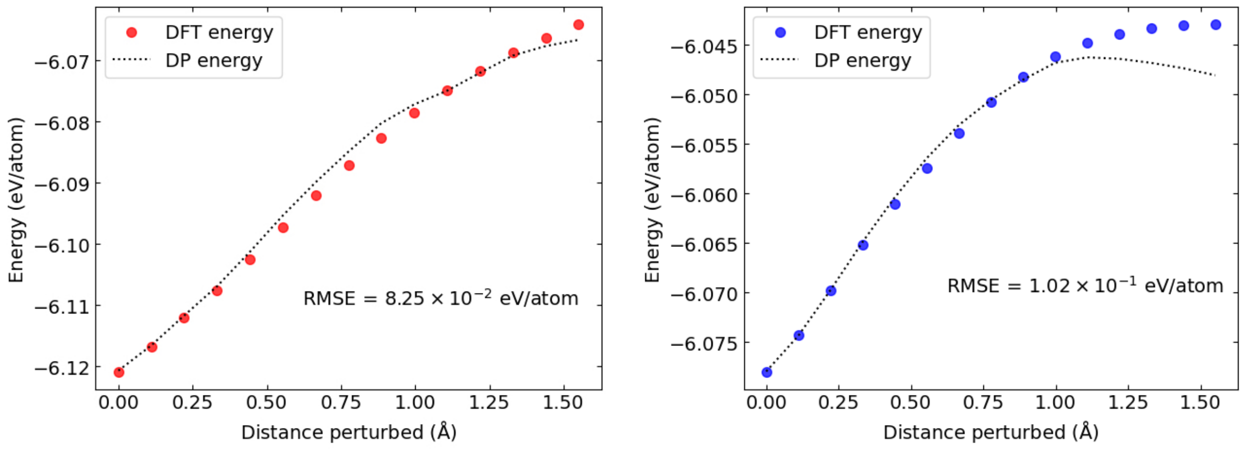

Figure S7: Bond dissociation energies calculated from DFT and the final DP for C–H (left) and C–NH_3 (right) bonds.

Proton Conductivity of Graphamine

We used our calculated values of D_S at different temperatures to estimate proton conductivities. These calculations used the Nernst-Einstein equation,

$$\sigma = \frac{F^2}{RT} c D_S, \tag{S2}$$

where F is the Faraday constant, R is the gas constant, T is the absolute temperature at which D_S is calculated, and c is the concentration of protons in the system. We estimated c, the number of moles of protons per volume, based on the p24C system. A single added proton corresponds to 1.67×10^{-24} moles of protons. The height of the graphamine cell after optimizing the c axis is about 6 Å. We have accounted for non-optimal layer packing by increasing the value of the c axis by 2 Å. This value is consistent with the approach used by Achar et al.[S6] in their calculations of the proton concentration for graphanol. While an expansion of 2 Å is somewhat arbitrary, it is physically reasonable, since larger expansion would be unlikely given the very strong van der Waals interactions between the graphamine layers. The cell lattice vectors (in Å) are (10.4, 0.0, 0.0), (-3.9, 6.8, 0.0), (0.0, 0.0, 8.0), which gives a volume of 5.6×10^{-22} cm^3. This results in a c value of 0.00292 mol/cm^3 for the p24C system. We note that increasing the lattice constant from 6 to 8 Å in the c axis decreases the concentration, and hence also reduces the conductivity, by 25%.

Mean Square Displacement Plots

D_S was computed using the mean square displacement plots by tracking the CEC in graphamine. Einstein's relation was used to estimate the ensemble average,

$$D_S = \frac{1}{2td} \langle \Sigma | r_{\text{CEC}}(t) - r_{\text{CEC}}(0)|^2 \rangle \tag{S3}$$

where t denotes the time, d denotes the dimensionality of the system, and r_{CEC} denotes the position of N atom having the proton. Since graphamine is a 2-D system, $d = 2$. We ran 20 independent 5 ns MD simulations for temperatures > 300 K to achieve sufficient statistical accuracy. For 300 K we ran 50 independent simulations for 10 ns to achieve the desired accuracy. The uncertainties of D_S are reported as twice the standard deviation values. We computed MSD from

$$\mathrm{MSD}(t) = \langle \Sigma |r_{\mathrm{CEC}}(t) - r_{\mathrm{CEC}}(0)|^2 \rangle \tag{S4}$$

using multiple time origins to improve the statistical accuracy. Note that a plot of $\mathrm{MSD}(t)/(4t)$ gives an estimate of D_S for $t \rightarrow \infty$ if it is approximately a straight line with zero slope. Plots of $\mathrm{MSD}/(4t)$ for the individual and averaged trajectories at each temperature are given in Figure S8.

Activation Energy for Proton Hopping

We have estimated the intrinsic activation energy for a single proton to hop from one NH_2 group to another, E_{hop}, using our final DP. We selected two images from a DP-MD simulation that involved a hopping event. We relaxed the initial and final images to their local minimia using the DP as a calculator. We then interpolated ten intermediate images between the starting and ending configurations and used these as initial guesses in the climbing image nudged elastic band (CI-NEB) method.[S7] The minimum potential energy surface for this specific proton hopping event is plotted in Figure 6 and graphics of the initial, transition, and final states are show in Figure S9. The forward barrier is 12.8 meV and the reverse barrier 21.3 meV, giving an average barrier of 17 meV. These barriers do not include zero point energy corrections. Including zero point energy corrections would likely lower the barrier, depending on the magnitude of the corrections at the initial, final, and transition states. We do not apply these corrections for two reasons. Firstly, standard generalized gradient corrected DFT is known to underpredict reaction barriers due to self-interaction errors.[S8]

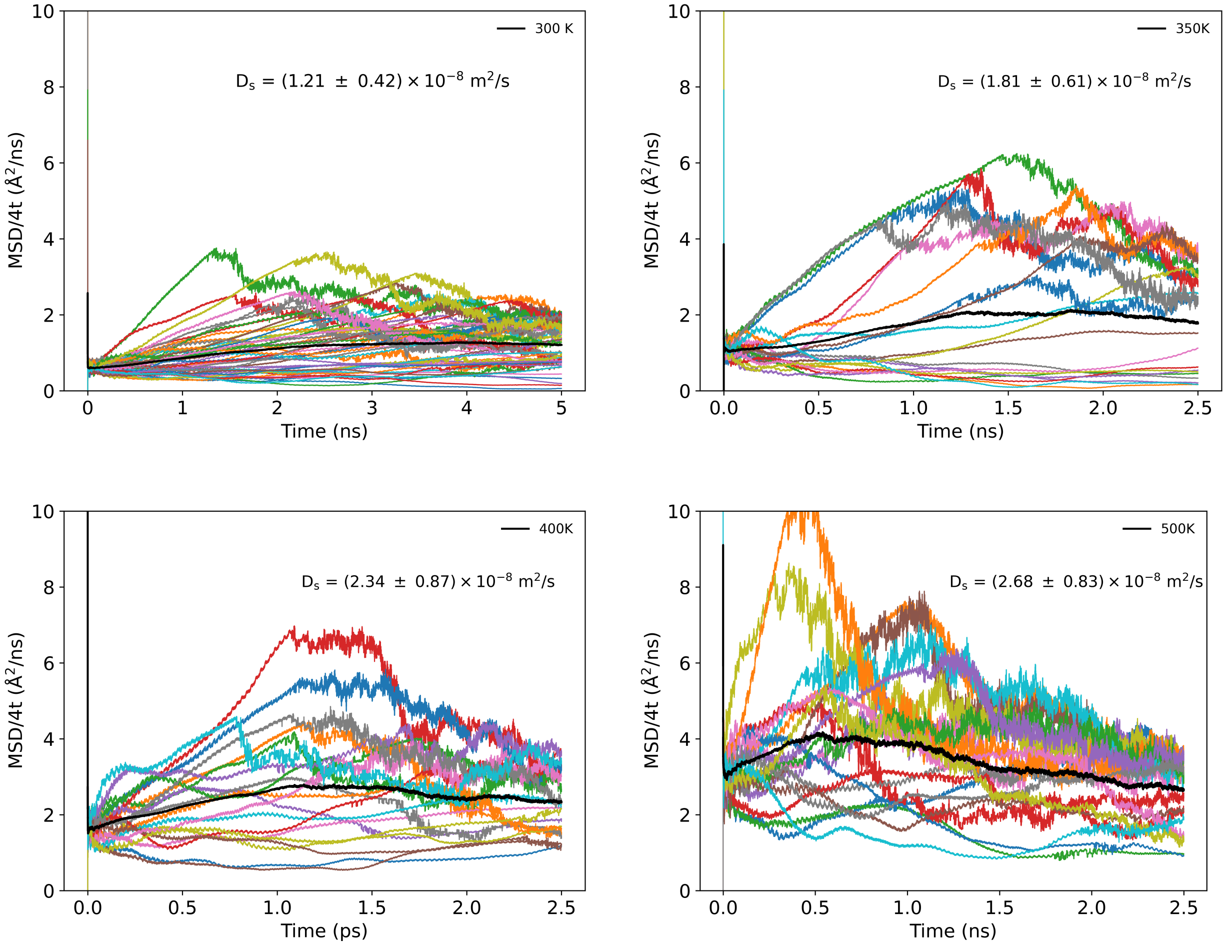

Figure S8: Individual and average MSD/$(4t)$ plots used for computing average diffusion coefficient values at different temperatures. The black line indicates the average MSD/$(4t)$.

Moreover, DFT has specifically been shown to underestimate proton hopping barriers in water compared with wavefunction methods.[S9] Therefore, including zero point energy corrections will likely lower a barrier that is already too low. Secondly, and more importantly, we are only interested in the magnitude of the barrier for a single hop compared with the apparent free energy barrier for diffusion, which we estimated to be 63 meV (see Figure 5). We deduce from these barriers that the single proton hop is obviously not the rate limiting step in proton diffusion.

The potential energy surface plotted in Figure 6 is just one possible pathway for a proton hop, given the large number of degrees of freedom for graphamine. Many other pathways having higher or lower barriers could be identified from other MD snapshots. To put this calculated barrier in context, we note that proton hopping between a water dimer in equilibrium is a barrierless process.[S10] It is therefore not unreasonable for an optimized isolated proton hop on graphamine to have a very low barrier. The key result is that proton diffusion in graphamine is not a result of near-equilibrium single proton hops, but a result of more complicated processes. Indeed, proton hopping at room temperature and above will not follow the minimum energy pathway, even for isolated proton hops because kinetic energy prohibits the system from being in a local minimum. It is likely that long-range proton hopping along Grotthuss chains at room temperature and above is facilitated by the librational motion of the NH_2 groups in the vicinity of the ${NH_3}^+$ moiety. The energy of the librational motion is much higher than E_{hop}, as can be deduced from the discussion on amine group rotations in the next section.

Amine Group Rotations

We plotted the x and y coordinates of hydroxyl hydrogens on graphanol and hydrogens on amine groups on graphamine using DP-MD simulations at 500 K for 10 ns. Part (a) in Figure S10 shows that there are complete rotations of hydroxyl groups in graphanol. In

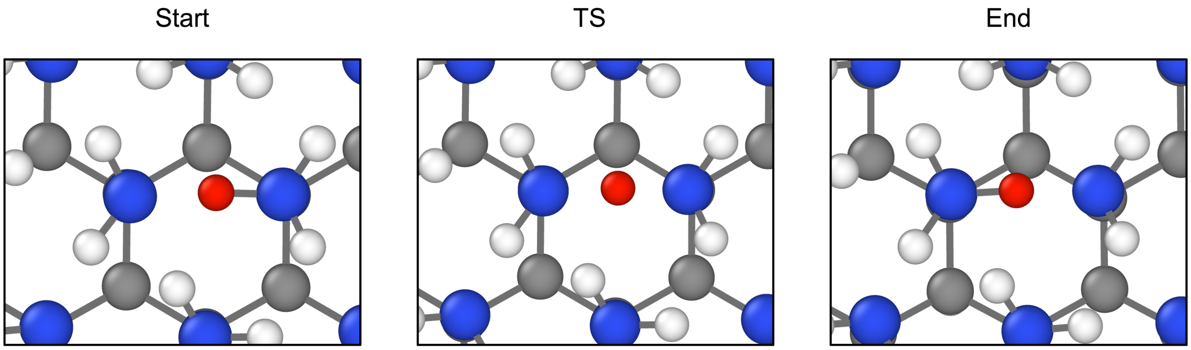

Figure S9: Graphics of the initial (Start), transition, (TS) and final (End) states, corresponding to images 0, 5 and 11, respectively, from Figure 6. Blue, N; gray, C; white, H; red, proton.

contrast, part (b) in Figure S10 depicts partial rotations, i.e., librational motion of amine groups in graphamine. This indicates that the amine groups in graphamine rotate much more slowly, with a significantly higher rotational barrier compared to graphanol.

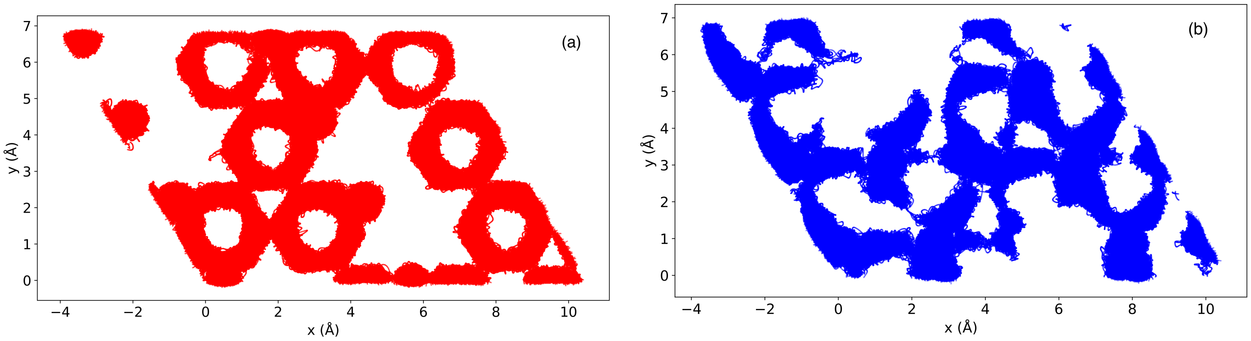

Figure S10: Scatter plots of x and y coordinates of (a) hydroxyl group hydrogens on graphanol and (b) amine group hydrogens in graphamine as calculated from 10 ns DP-MD simulations at 500 K.

Proton Transport Mechanism

We used the unwarpped coordinates written at the end of the CEC calculations at 600 K to make spider plots. We plot y-coordinates vs the x-coordinates of the N atom that is labeled as a CEC. We counted edges for each node and have marked nodes having 3, 4, 5 or 6 edges for graphamine in Figure S11 and for graphanol in Figure S12.

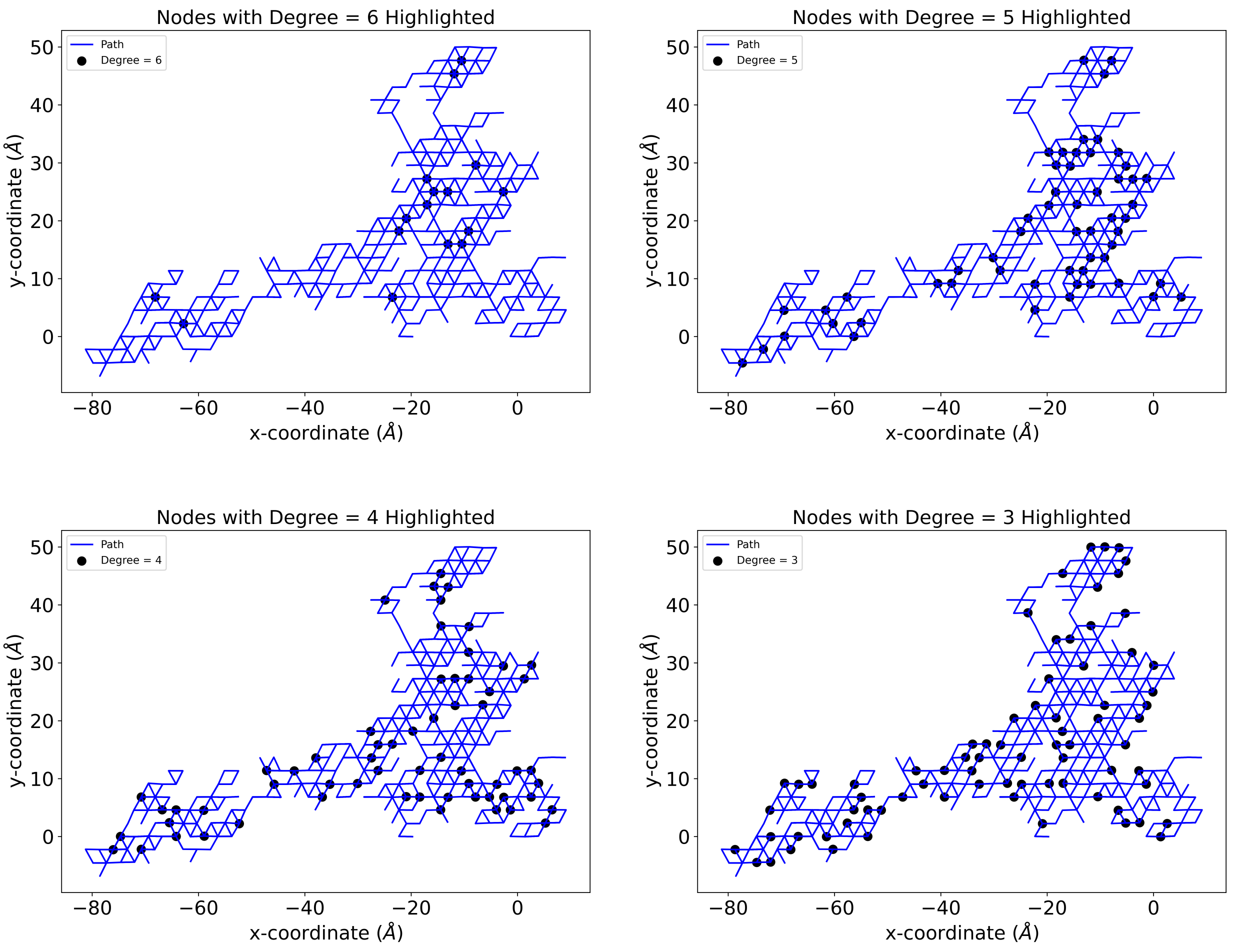

Figure S11: Spider plots for graphamine at 600 K marking nodes having 6, 5, 4, or 3 edges.

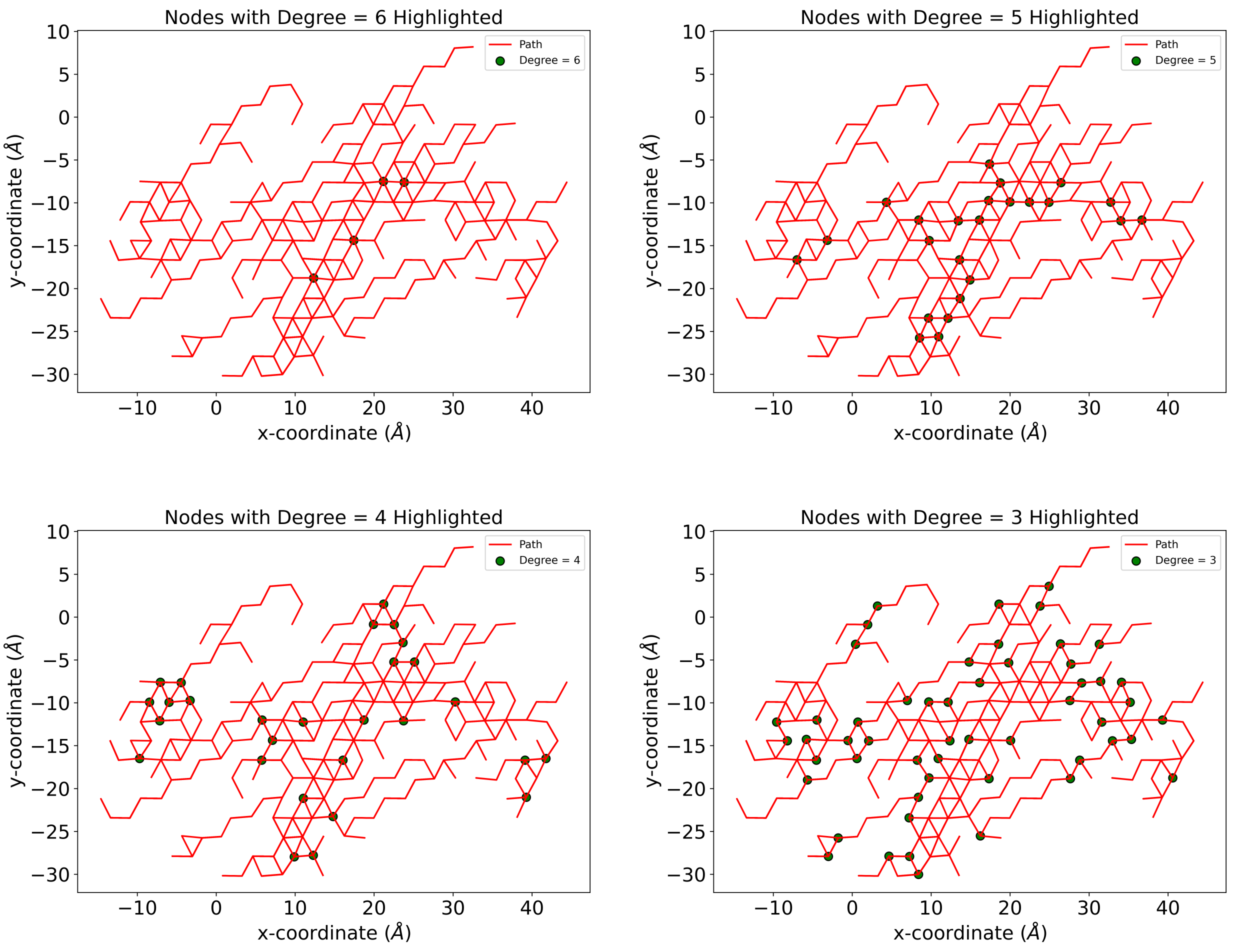

Figure S12: Spider plots for graphanol at 600 K marking nodes having 6, 5, 4, or 3 edges.

References

(S1) Wang, H.; Zhang, L.; Han, J.; Weinan, E. DeePMD-kit: A deep learning package for many-body potential energy representation and molecular dynamics. *Comput. Phys. Commun.* **2018**, *228*, 178–184.

(S2) Zeng, J.; Zhang, D.; Lu, D.; Mo, P.; Li, Z.; Chen, Y.; Rynik, M.; Huang, L.; Li, Z.; Shi, S. et al. DeePMD-kit v2: A software package for deep potential models. *J. Chem. Phys.* **2023**, *159*.

(S3) Zhang, Y.; Wang, H.; Chen, W.; Zeng, J.; Zhang, L.; Wang, H.; others DP-GEN: A concurrent learning platform for the generation of reliable deep learning based potential energy models. *Comput. Phys. Commun.* **2020**, *253*, 107206.

(S4) Togo, A.; Chaput, L.; Tadano, T.; Tanaka, I. Implementation strategies in phonopy and phono3py. *J. Phys. Condens. Matter* **2023**, *35*, 353001.

(S5) Togo, A. First-principles Phonon Calculations with Phonopy and Phono3py. *J. Phys. Soc. Jpn.* **2023**, *92*, 012001.

(S6) Achar, S. K.; Bernasconi, L.; DeMaio, R. I.; Howard, K. R.; Johnson, J. K. In silico demonstration of fast anhydrous proton conduction on graphanol. *ACS Appl. Mater. Interfaces* **2023**, *15*, 25873–25883.

(S7) Henkelman, G.; Uberuaga, B. P.; Jónsson, H. A climbing image nudged elastic band method for finding saddle points and minimum energy paths. *J. Chem. Phys.* **2000**, *113*, 9901–9904.

(S8) Shukla, P. B.; Mishra, P.; Baruah, T.; Zope, R. R.; Jackson, K. A.; Johnson, J. K. How Do Self-Interaction Errors Associated with Stretched Bonds Affect Barrier Height Predictions? *J. Phys. Chem. A* **2023**, *127*, 1750–1759, PMID: 36787213.

(S9) Sadhukhan, S.; Muñoz, D.; Adamo, C.; Scuseria, G. E. Predicting proton transfer barriers with density functional methods. *Chem. Phys. Lett.* **1999**, *306*, 83–87.

(S10) Geissler, P. L.; Van Voorhis, T.; Dellago, C. Potential energy landscape for proton transfer in (H2O)3H+: comparison of density functional theory and wavefunction-based methods. *Chem. Phys. Lett.* **2000**, *324*, 149–155.
